# Subject-matter and intensional operators I: conditional-agnostic analytic implication

**DOI:** 10.1007/s11098-023-01952-4

**Published:** 2023-04-29

**Authors:** Thomas Macaulay Ferguson

**Affiliations:** 1 ILLC, University of Amsterdam, Amsterdam, Netherlands; 2 Arché Research Centre, University of St. Andrews, St. Andrews, Scotland

**Keywords:** Subject-matter, Topic-sensitive intentional modals, Analytic implication

## Abstract

Although logical settings are typically concerned with tracking *alethic* considerations, frameworks exist in which *topic-theoretic* considerations—e.g., tracking *subject-matter* or *topic*—are given equal importance. Intuitions about extending topic through a propositional language are generally straightforward for *extensional* cases. For a number of reasons, arriving at a compelling account of the subject-matter of *intensional operators*—such as intensional *conditionals*—is a more difficult task. In particular, the framework of *topic-sensitive intentional modals* (TSIMs) championed by Francesco Berto and his collaborators leave the topics of intensional formulae *undefined*, which artificially constricts the expressivity of the theory. This paper proposes an approach to fill in this lacuna, emphasizing an analogous problem in Parry-style containment logics. In this setting, the approach receives a proof-of-concept through the introduction of a natural and general family of subsystems of Parry’s ${\textsf{PAI}}$—with sound and complete axiomatizations—that allow a fine degree of control over the topics of intensional conditionals.

## Introduction

Despite the traditional slogan that logic is “topic-neutral,” treating the semantic feature of subject-matter as a logically significant feature has a long history. For example, the eponymous feature of *relevance* central to relevant logic (Anderson et al. 1975 or Routley et al. 1982) can be understood as a requirement that the topics of antecedents and consequents of valid conditionals *overlap*. A refinement of this intuition of relevance that has intermittently appeared is that validity requires not only *overlap* but *inclusion* of the topic of the consequent within that of the antecedent.

The first full-throated development of this intuition is due to William Parry, who introduced a logic of *analytic implication* in Parry (1933), inspired by a thesis that if validity is to be *analytic* the subject-matter of the consequent cannot *exceed* that of the antecedent. A good illustration of Parry’s motivations is the following:

If a system contains the assertion that two points determine a straight line, does the theorem necessarily follow that either two points determine a straight line or the moon is made of green cheese? No, for the system may contain no terms from which ‘moon,’ etc., can be defined. (Parry, 1968, p. 151)

Although this paper largely works within the setting of Parry’s analytic implication, the *ultimate* target is a more recent logical framework in which the inclusion of subject-matter is taken seriously: The theory of *topic-sensitive intentional modals* (TSIMs) developed and championed by Francesco Berto and his collaborators in e.g. Berto (2019b, 2022).

The eponymous TSIMs are two argument operators *X* producing formulae of the form $X^{\varphi }\psi$. Where *X* is interpreted as some type of intentional mental state, the intended interpretation of the formula is “Given $\varphi$, an agent *X*s that $\psi$.” Particular applications are abundant and wide-ranging; *X* can be understood as *knowability relative to information* (see e.g. Berto & Hawke, 2021), as *static belief revision* (see Berto, 2019a), or *mental simulation* (see Berto, 2018). Formally, TSIMs are a pairing of variably strict conditionals (in the style of e.g. Lewis, 1973) with a topic inclusion filter. The latter condition imposes a requirement on the truth of $X^{\varphi }\psi$ that the topic of $\psi$ is included in the topic of $\varphi$.

A topic filter presupposes that topic has been defined for all formulae, including those including an intensional operator. However, in presentations like Berto’s—or related formalisms like those employed in containment logics—the topic for such intensional formulae is either undefined or recognizably inadequate. This paper is the first in a sequence of investigations into the formal representation of the topic or subject-matter of intensional operators or connectives in a formal language. The starting point is the sketch outlined in Ferguson (2021).

### The problem

The current state of the art of TSIMs faces limitations concerning the assignment of topics to an intensional sentence $\varphi$: presentations invariably either suffer from *inadequate refinement* for coarsely assuming $\varphi$ to be topic-transparent or suffer from *inadequate expressivity* for declining to assign a topic to $\varphi$.

Now, to consider the former case of inadequate refinement, let us consider some of the formalism in more detail. The model theory of TSIMs incorporates a *join semilattice* $\langle \mathcal {T},\oplus \rangle$ understood as a domain of *topics*; a function *t* is responsible for assigning topics to formulae. Two important features are reflected in the recursive clauses for *t* relating the topic of complexes to the topics of their parts; Berto describes these assumptions in Berto (2022):

- **Negation Transparency:** $${t}(\lnot \varphi )={t}(\varphi )$$
- **Junctive Transparency:** $${t}(\varphi \wedge \psi )={t}(\varphi \vee \psi )={t}(\varphi )\oplus {t}(\psi ).$$

In many cases, the conditions seem fine; the interchangeability between e.g. conjunctions and *commas* in many sequent calculi does suggest a reading in which conjunction (and thus disjunction) is nothing more than *punctuation*. To make the additional assumption of synonymy of  and $\lnot \varphi \vee \psi$ will commit oneself to *Junctive Transparency* holding of the material implication  as well. But not all syncategorematic terms are as topic-theoretically inert.^1^

In the particular presentation of *knowability relative to information* (${\textsf{KRI}}$) described in Berto and Hawke (2021), however, topics for not only TSIMs but also the strict implication  are transparent. This directly leads to some internal tension, as we can observe. Read “$\textsf{K}^{\varphi }\psi$” as “relative to information $\varphi$, it is knowable that $\psi$.” Then:

#### Observation 1

In $\textsf{KRI}$, the inferenceis valid.

In other words, if $\psi$ is knowable given information $\varphi$, then $\varphi$ supports the *strict implication*  as well.

But this result is thematically at odds with the central property of *nonmonotonicity* championed in Berto and Hawke (2021). To borrow an example, suppose that it holds that:

1. Given that *The Times* reported that Manchester United won the match, one is in a position to know that Manchester United won the match.

By the lights of Observation 1, the inference from **[1]** to the below **[2]** must hold:

2 Given that *The Times* reported that Manchester United won the match, one is in a position to know that that *The Times’* reporting of Manchester United’s winning *necessarily entails* that Manchester United wins.

The only case in which an inference from **[1]** to **[2]** would be plausible were if *The Times*’ accuracy were sufficient to act as a metaphysical guarantor. But this would place us at odds with the very grounds for desiring nonmonotonicity, namely, that information is not always reliable.

In presentations of the latter type of inadequate inexpressivity (like Berto 2022), the function *t* assigning topics to formulae is *partial*; while the topics of all *extensional* formulae are defined, for intensional formulae—i.e., those in which a strict implication or TSIM appears—the topic is *undefined*. Unlike extensional connectives, which contribute to topic no more than punctuation marks, intensional operators seem to exert a *transformational* influence on the subject-matter of complexes, ruling out naive alternatives in which intensional operators are *topic-transparent*.

This greatly curtails the scope and ultimately limits the utility of the framework. Consider the setting of *acts of imagination* developed in Berto (2018) and its representation in the context of TSIMs in Berto (2019b). In practice, agents’ imaginative activity frequently includes intensional notions (*e.g*, one can imagine that one knows some proposition). Without an account of the topic of the intensional *counterfactual*, a relatively simple truth

3 In an act of imagination in which ‘had Sam gone home, she would have eaten dinner and gone to bed’ is true, one also imagines that ‘had Sam gone home, she would have eaten dinner’.

Cannot be evaluated.

Similarly, return to the context of knowability relative to information described in Berto and Hawke (2021); not only is *extensional* information knowledge-constitutive, but *intensional* information—e.g. information expressing the metaphysical necessity of such-and-such a proposition—can serve to ground knowability. In other words, to capture the full breadth of knowability relative to information, a means should exist to evaluate sentences such as:

4 Given the information that it is necessary that water is $H_{2}O$, one is in a position to know water is $H_{2}O$.

If the framework is to reach its full realization, it must be faithful to linguistic practice. If the framework is to be faithful to linguistic practice, the assignment of formulae to topics must not only be *total* but be *extensible* to a wide variety of intensional operators. Thus, we have a need to introduce a framework for the assignment of topics to intensional formulae.

### The general proposal

Our proposal is a modest one. Even if the topic of an intensional conditional is *compositional*, one can imagine numerous ways in which the topic of any *particular* intensional conditional may differ from the mere *fusion* of the topics of its subformulae. Likewise, one can imagine that distinct types of intensional conditionals determine their topics in different ways. *E.g.*, while an $\textsf{S5}$ strict conditional is arguably *about* the constituents of the actual world, the nature of a *counterfactual* conditional seems to require that its topic *omits* these inhabitants from its subject-matter. Given the justified true belief account, the topic of a TSIM modeling *knowledge* may include that of the corresponding TSIM modeling *belief*. Given the variety of intensional operators—and number of distinct topic-theoretic relationships holding between them—an appropriate general machinery should be weak enough to cover a maximal number of cases.

Ways in which we might characterize the topic-theoretic behavior of an intensional conditional $\rightarrow$ include:

- cases in which ${t}(\varphi \rightarrow \psi )$ arguably *properly extends* the fusion of ${t}(\varphi )$ and ${t}(\psi )$ (considered in Sect. 2.1)
- cases in which ${t}(\varphi \rightarrow \psi )$ is arguably *incommensurable* with the fusion of ${t}(\varphi )$ and ${t}(\psi )$ (considered in Sect. 2.2)
- cases in which the topics ${t}(\varphi \rightarrow \psi )$ and ${t}(\psi \rightarrow \varphi )$ may differ from one another (considered in Sect. 2.3).

While compositional, different types of intensional conditionals may determine different accounts of topic; e.g., the topics of $\varphi$ and $\psi$ may bear one relationship to that of a counterfactual  and another to a strict conditional . In other words, one size need not fit all. The proposal is therefore guided by a simple agnosticism. We wish to provide a framework that is sufficiently *permissive* to not force a theory of topic upon a conditional when unwarranted; but also demand of the framework that it be sufficiently *modular* to allow the representation of stronger, more detailed theories of topic corresponding to individual types of intensional conditionals.

To capture this intuition, we equip the topic semilattice with a binary function $\multimap$ and interpret the topic ${t}(\varphi \rightarrow \psi )$ as ${t}(\varphi )\multimap {t}(\psi )$. Beyond its being a function, nothing else is assumed; however, various theses about the topic of a given intensional conditional can be captured by *imposing* new constraints on the properties of $\multimap$.

This agnosticism is thus recognizably a *feature* rather than a bug. Just as Kripke models are agnostic with respect to the model-theoretic characterization of *necessity*—$\textsf{K}$ assumes little about its accessibility relation *R*—so, too, shall we remain agnostic about the topic-theoretic characterization of intensional sentences. But the proposal is not *vacuous*; as we will discover, there is enough subtlety to allow a fine degree of control over allowing the representation of various intuitions about the behavior of the topics of such sentences.

## Topic and intensional conditionals

In the foregoing section, we briefly considered several dimensions along which the topic of an intensional conditional could differ from the fusion of the topics of its parts. In this section, we will examine each of these cases more closely.

### Ampliativity

Many types of intensional conditionals may be considered to be *topic-theoretically ampliative*, i.e., the implication operator plays a role in determining the overall subject-matter of the complex, whether the influence is *additive* or *transformative*. The most direct route to intensional conditionals’ ampliativity begins by taking note of a familiar characterization: it is common to describe intensional conditionals as expressing a *relation* between antecedent and consequent. The interpretation of conditionals as expressing a relation—of *some* kind—between statements is a well-established and frequently encountered idiom, pronounced not only in revisionary, intensional projects like Lewis and Langford (1959) or Routley et al. (1982) but also in mainstream, classical settings like Quine (1981).

It is generally acknowledged (e.g., Yablo, 2014, Berto, 2022, Fine 2020) that a predicate makes a prominent contribution to the subject-matter of a sentence. Suppose one is told the following:

5 *A* logically entails *B*.
6 *A* increases the likelihood of *B*.

It is unobjectionable to say that **[5]** is *about* something different than **[6]**. The item about which their subject-matter most clearly diverges is arguably the *central relation*.

Conventionally, an atom *Rab* is about the individuals *a* and *b* and the relation *R* that holds between them. If intensional connectives likewise express relations *between statements* then to say that the relation $\varphi \rightarrow \psi$ is about the propositions $\varphi$ and $\psi$ and the entailment relation that holds between them is structurally analogous. If this is insufficient, one can make it *ontologically* or *notationally* analogous as well.

So I am suggesting that in some cases intensional conditionals contribute something to the overall subject-matter in virtue of their expressing a *relation*. But there is a potential trap here. That the material implication, too, expresses a relation is very nearly as common a theme as intensional implication. Even those—like Routley et al. (1982)—who are overtly hostile to the material conditional, nevertheless concede its status as a relation, albeit a *useless* relation.

If the material conditional *is* a relation, we may have a commitment to its subject-matter’s reflection in the subject-matter of the complex. Nevertheless, the material conditional depends *exclusively* on the truth values of its subformulae. One might argue that its topic is exhausted by the corresponding truth-function, leaving the subject-matter of the material conditional as no more topic-laden than a conjunction. Thus, one can maintain that the material conditional contributes to the subject-matter of the complex, while thinking that it respects *Junctive Transparency*: Like intensional conditionals, the corresponding relation is reflected in the overall subject matter; unlike intensional conditionals, its contribution is *vacuous*. So if *Junctive Transparency* fails to reflect the true *structure* of an indicative conditional, the subject-matter that it ultimately *assigns* will always be correct.

### Incommensurability

If the discussion in Sect. 2.1 establishes cases in which the topic of an intensional conditional can *exceed* that of the fusion of the topics its parts, there are also cases in which the conditional’s topic may be *exceeded by* the fusion. Although tentative, there is some evidence that this may occur in the case of *counterfactual conditionals*.

To provide an informal example of when this *incommensurability* may appear, recall two facts: First, the invention of *bifocal lenses* is commonly attributed to the American Benjamin Franklin. Second, the French physicist Augustin-Jean Fresnel—who worked in optics contemporaneously with Franklin—was the child of Jacques Fresnel. With these names introduced, consider the following sentence:

7 The inventor of bifocals was the child of Jacques Fresnel.

Although **[7]** is a false statement, there is a straightforward—if informal—sense in which it is a falsehood *about* Benjamin Franklin. Arguably, this is because at the *context of evaluation*—namely, the actual world—the definite description “the inventor of bifocals” refers to Franklin. But consider the conditional:

8 Had the inventor of bifocals been the child of Jacques Fresnel, then their invention would be hailed as a feat of French ingenuity.

As **[8]** is a *counterfactual*, its evaluation ranges over only worlds in which the antecedent is true, and thus omits the actual world from contexts in which it is evaluated. Moreover, insofar as it is *metaphysically impossible* that Benjamin Franklin could have been the child of Jacques Fresnel, at no context of evaluation *could* Franklin satisfy the definite description “the inventor of bifocals.” Consequently, **[8]** is not *about* Benjamin Franklin at all. *If* **[7]** is about Franklin—whether in whole or in part—then some element of subject-matter that present in the subformula would be *omitted* from the subject-matter of **[8]**.

As a referee has generously pointed out, this example may rely on an equivocation between senses of “about.” Recalling Yablo’s Wildean insight that “the Queen is not a subject” (Yablo, 2014, p. 26), the topics **Franklin** and **inventor of bifocals**—the subject-matters of the referring terms “Benjamin Franklin” and “inventor of bifocals,” respectively—may be distinct in spite of their being codenotational. I acknowledge that this is a real problem for the example.

Nevertheless, there is reason to think that in spite of this distinctness there is some common part of the subject-matters **Franklin** and **inventor of bifocals**. Two individuals can, after all, coordinate the topics of discourses in cases in which each uses a different referring term. This is explicitly sketched in the concluding pages of Hawke (2018), whose issue-based theory would treat Franklin as a common constituent of the terms’ subject-matter, albeit under distinct Salmon-style guises. Whatever this shared topic part might be, it would certainly be lacking in the subject-matter of **[8]**.

The state of research into the topics of definite descriptions may yet be too young to lend itself to a knock-down argument either way. But pressing the issue requires only showing cause to exercise caution before endorsing commensurability. After all, there are numerous contexts in which the transformative qualities of intensional or intentional operators challenge this thesis of subtopic preservation in all contexts, i.e., that topics of the parts are by necessity preserved as subtopics of a complex. Following Berto’s example of attitude ascriptions in Berto (2022, p. 65), the subject-matter of “Mary believes that Scotland is lovely” is not necessarily about Scotland, but rather about a proposition. Yablo’s remarks on Hempel-style “all nonblack things are nonravens” suggest that the complex is *not* about ravens despite a subsentence thereof being about ravens. Such cases strike me as sufficiently hazy to give one pause, at least.

This should suffice insofar as my aims are ecumenical. The framework described in the next section is modular enough to allow cases of incommensurability. If one accepts that there is reason to be cautious, one can model situations in which subtopic preservation fails. If one accepts instead the thesis of subtopic preservation, then one may elect to employ a modest extension of the following system in which commensurability is guaranteed, an extension which conveniently corresponds to the reintroduction of one of Parry’s original axioms. We will return to this extension and its metatheory in Sect. 4.4.

### Order-sensitivity

When considering the subject-matter of *first-order predicates*—i.e., relations holding between *individuals*—it is relatively uncontroversial to suppose the *order in which arguments appear* is a determinant in the assignment of the subject-matter of a literal. One finds this codified in e.g. Berto (2022) in the explicit *rejection* of the following thesis:

$$\begin{aligned} {\textbf {Constituent Equivalence:}}\,{{t}}(Rab)={{t}}(Rba) \end{aligned}$$

Yablo’s remark in Yablo (2014) makes a compelling, if informal, argument against *Constituent Equivalence*, asking:

Why is man bites dog a better headline than dog bites man? One thing we can certainly say is that it is on a more interesting topic. A more interesting topic is a different topic. Yablo (2014)

Although the remark invokes *first-order relations* to drive the example, Yablo’s underlying intuitions are equally sound with respect to *relations in general*.

As we’ve mentioned, *intensional* conditionals are frequently acknowledged to *express a relation*—e.g., an *entailment relation*—between antecedent and consequent. (In contrast, *truth-functional* material conditionals either fail to express a relation or, if they do, express only a vacuous relation impoverished of subject-matter.) Such *relations between propositions* are as authentically relational as first-order *relations between individuals*. The implausibility of *Constituent Equivalence* in the case of intensional conditionals is illustrated by the severity of the incongruity between the *topics* of the following two conditionals:

9 Were John to win the lottery, he would retire tomorrow.
10 Were John to retire tomorrow, he would win the lottery.

Intuitively, **[9]** and **[10]** paint radically different pictures. The former is an unsurprising statement about the actions the subject John would take in case of a sudden windfall. In contrast, the latter sentence expresses an *unusual* claim about John—retirement is an event that most people reach *without* a windfall, so John must have some *surprising* quality that ensures a lottery win upon his retirement. Like Yablo’s first-order example, **[10]** is clearly about a more *interesting* topic than **[9]** and, consequently, a *different* topic.

We can strengthen this. If one adopts a view like that of Kratzer in (1991) in which the role of the antecedent (the “if-clause”) is that of a *restrictor*, i.e., the antecedent serves to restrict the set of worlds or states against which the consequent is to be evaluated, then this can be taken a step further. In **[9]**, there is a sense in which a class of scenarios in which John wins the lottery is not only part of how to determine the *truth* of **[9]**; these states are part of what the sentence is *about*.

To illustrate, consider a conversation in which one participant asks, “What do you think would happen if John were to retire tomorrow?” Despite an *a priori intersection* between topics, an interlocutor who utters **[9]** has nevertheless veered *off-topic* in a way that an utterer of **[10]** does not. **[10]** *addresses the question*—if perhaps unrealistically—in virtue of its being *about* those situations in which John retires. **[9]**, on the other hand, fails to reflect the restriction implicit in the question; it is *primarily* about lottery-winnings and only *subordinately* about retirements.

This only makes sense in case the intensional conditionals in **[9]** and **[10]** nontrivially influence their respective topics.

## The content of intensional conditionals in analytic implication

The prototypical formal treatment of a deductive system in which subject-matter is taken seriously is doubtlessly William Parry’s logic of *analytic implication* $\textsf{PAI}$, first introduced in Parry (1933). Parry’s logic is motivated by a desire to give a formal account of entailment as drawing the consequent from the antecedent through a process of *analysis*. As discussed in Ferguson (2017), a guiding intuition is that a necessary condition for the *analyticity* of an entailment relation is that the consequent of a valid entailment should not *include subject-matter* (or *concepts*) *not present* in the antecedent. The definitive statement of this condition is the *Proscriptive Principle* that

No formula with analytic implication as main relation holds universally if it has a free variable occurring in the consequent but not the antecedent. (Parry, 1968, p. 151)

Parry’s approach has had its share of criticism (Kielkopf, 1975; Anderson et al., 1975; Routley et al., 1982) which have been taken up elsewhere (Ferguson, 2015). Regardless of such criticism, Parry’s $\textsf{PAI}$ enjoys some intuitive appeal.

Additionally, the topic-theoretic device employed in Kit Fine’s model theory for Parry’s analytic implication is virtually identical to that employed in the TSIM framework. Results from one setting may transfer nearly immediately to the other. Thus, we will first consider the proposal in the context of $\textsf{PAI}$. The environment of Fine’s model theory is very natural and provides a suitable test-bed to explore different ways to assign subject-matter to intensional conditionals, but this is merely a convenience. As I will point out at many points in the sequel, nothing essentially hinges on this interpretation. Modifying the models slightly will allow the representation of other intensional conditionals in its stead.

### The Parry analysis

Let $\mathcal {L}$ be a propositional language including negation ($\lnot$), conjunction ($\wedge$), disjunction ($\vee$), and an intensional conditional ($\rightarrow$). I informally refer as well to the material conditional  defined from $\lnot$ and $\vee$ in the standard way. The starting point for the structures described in this paper is Fine’s semantics for Parry’s logic and the default interpretation of $\rightarrow$ will be the analytic implication connective.

The initial semantic analyses of Parry’s logic began by examinations of related systems by Dunn (1972) and Urquhart (1973). The first full model theory for Parry’s logic itself was provided by Fine (1986).^2^ Fine’s semantics for Parry’s system equips each world *w* of an $\textsf{S4}$ Kripke model with join semilattices of *topics* $\langle \mathcal {T},\oplus \rangle$.^3^

#### Definition 1

A $\textsf{PAI}$ Fine model is a tuple $\langle W,R,\mathcal {T},\oplus ,v,{t}\rangle$ with:

- $\langle W,R\rangle$ is an $\textsf{S4}$ Kripke frame
- For each $w\in W$, $\langle \mathcal {T}_{w},\oplus _{w}\rangle$ is a join semilattice
- *v* is a valuation from atomic formulae to *W*
- For each $w\in W$, ${t}_{w}$ is a function mapping atomic formulae to $\mathcal {T}_{w}$
- If $wRw'$ and $t_{w}(p)\le _{w}t_{w}(q)$ for atoms *p*, *q* then $t_{w'}(p)\le _{w'}t_{w'}(q)$.

As a join semilattice, each $\langle \mathcal {T}_{w},\oplus _{w}\rangle$ defines a partial order $\le _{w}$ so that $a\le _{w}b$ if $a\oplus _{w}b=b$. Moreover, the constraint guaranteeing that topic inclusion between atoms across accessible worlds ensures the persistence of topic inclusion for any sentences.

These join semilattices reflect an intuition that topics can be *fused* together. The functions ${t}_{w}$ are responsible for assigning topics to formulae; for $\textsf{PAI}$, the assignment is determined as follows:

#### Definition 2

The topic assignment function ${t}_{w}$ is extended through the language:

- $${t}_{w}(\lnot \varphi )={t}_{w}(\varphi )$$
- ${t}_{w}(\varphi \star \psi )={t}_{w}(\varphi )\oplus _{w}{t}_{w}(\psi )$ for binary connectives $\star$.

Truth at a world is defined:

#### Definition 3

Truth conditions are defined recursively:

- $$w\Vdash p$$ if $$w\in v(p)$$
- $$w\Vdash \lnot \varphi $$ if $$w\nVdash \varphi $$
- $$w\Vdash \varphi \wedge \psi $$ if $$w\Vdash \varphi $$ and $$w\Vdash \psi $$
- $$w\Vdash \varphi \rightarrow \psi $$ if $${\left\{ \begin{array}{ll} \text{ for } \text{ all } w' \text{ such } \text{ that } wRw'\text{, } \text{ if } w'\Vdash \varphi \text{ then } w'\Vdash \psi \\ {t}_{w}(\psi )\le _{w}{t}_{w}(\varphi ) \end{array}\right. }$$.

Given truth conditions for negation and conjunction, those for disjunction ($\vee$) and material implication () can be inferred from the above.

Note that the “double-barreled” nature of the truth conditions for analytic implication reveals it to presuppose a “two-component” approach to semantic content in the sense of Berto et al. (2019). The valuation function *v* and topic-assignment function *t* each serve as an independent component that mutually determine content.

Its topic assignment function encapsulates what might be considered a property of *topic vacuity* of the intensional conditional, i.e., that the intensional conditional connective itself contributes nothing to—and exerts no influence over—the overall subject-matter of a sentence.

In Sect. 2.1, I identified cases in which the influence of *some* intensional conditionals on the overall subject-matter of a complex is *ampliative*, which suggests that subject-matters of intensional conditionals may, in general, be distinct from the fusion of the subject-matters of their components. In the case of such topic-ampliative conditionals, the topic vacuity of *t* in Definition 1 is recognizably deficient. Something more subtle is required.

But there is a further step in the development of the topic-theoretic apparatus of $\textsf{PAI}$ that is worth mentioning refined enough to handle this ampliativity. Thus, we examine a refinement of $\textsf{PAI}$ in which a slightly more nuanced picture is described.

### Fine’s revisions

In the concluding pages of Fine (1986), Fine pauses to consider some potential elaborations on his model theory for Parry’s system, identifying in particular some discomfort with applying *Junctive Transparency* to Parry’s intensional conditional $\rightarrow$.

Fine observes that Parry offers the Proscriptive Principle as a thesis about the inclusion of *concepts* (*Begriffe*) while simultaneously describing analytic implication itself as a concept (*Begriff*):

Another change, suggested by Parry [in Parry (1933)], arises from treating analytic implication as a concept. No proposition not containing this concept could then analytically imply a proposition containing that concept. (Fine, 1986, p. 177)

Apparently, this suggestion is not explicitly posed by Parry; rather, the modification sketched by Fine has its origins in Parry’s choice of terminology:

[T]he concept (*Begriff*) of logical consequence should have the specified property, since the conclusion cannot contain any concepts (*Begriffe*) other than the premises.^4^ (Parry, 1933, p. 5)

In other words, if the Proscriptive Principle proscribes the introduction of new concepts (*Begriffe*) and analytic implication is *itself* a concept (*Begriff*), then the proscription ought to extend to preclude the introduction of *this* concept as well.

Having acknowledged that relations expressed by intensional conditionals may have an ampliative or transformational effect on component subject-matter, Fine’s suggestion is clearly a step in the right direction. Conditionals—most importantly, *intensional* conditionals—are often described as asserting that an entailment *relationship* holds between two propositions, however. Consequently, there seem to be cases in which the appearance of $\rightarrow$ in $\varphi \rightarrow \psi$ makes a similar *contribution* to the complex sentence’s subject matter as the predicate “is married to” contributes to the subject-matter of “Jill is married to Pat.”

The recipe to revise to the model theory that follows from Fine’s observation is straightforward; for each semilattice $\langle \mathcal {T}_{w},\oplus _{w}\rangle$, we must ensure that there is a concept $\mathfrak {i}_{w}$ (for “implication”) that stands in for the contribution made by analytic implication itself. Described in Ferguson (2021) as the *coarse* proposal, let us follow Fine in Fine (1986) in defining a *revised* $\textsf{PAI}$ model:

#### Definition 4

An $\textsf{R}/\textsf{PAI}$ Fine model is a tuple $\langle W,R,\mathcal {T},\oplus ,v,{t}\rangle$ differing from Definition 1 by including for each $w\in W$ a privileged element $\mathfrak {i}_{w}\in \mathcal {T}_{w}$ such that ${t}_{w}$ is extended as follows:

- $${t}_{w}(\lnot \varphi )={t}_{w}(\varphi )$$
- ${t}_{w}(\varphi \star \psi )={t}_{w}(\varphi )\oplus _{w}{t}_{w}(\psi )$ for extensional connectives $\star$
- $${t}_{w}(\varphi \rightarrow \psi )={t}_{w}(\varphi )\oplus _{w}\mathfrak {i}_{w}\oplus _{w}{t}_{w}(\psi )$$.

Continuity with $\textsf{PAI}$ models is reinforced by retaining the truth conditions outlined in Definition 3.

Fine does not provide an axiomatization of the suggested modification to the model theory. It may be of historical interest to do so, but the matter is not taken up here.^5^ Our interest in describing Definition 4 is in its role as an intermediate waypoint during the maturation of Parry’s analytic implication into the more refined alternative we will consider in the sequel. Insofar as Definition 4 makes space for the phenomenon of *ampliativity* considered in Sect. 2.1, it undoubtedly improves on Definition 1.

But identifying the models as an intermediate position indicates that there are drawbacks. These rest on the coarseness of the proposal and what may be thought of as *perfunctory contribution* made by the intensional conditional. A notable consequence of the coarse proposal is clear. According to Definition 4, an intensional conditional’s contribution to the subject-matter of a complex is determined entirely by the binary dimension of its appearance—or not—in the complex. Its contribution is ignorant of other *prima facie* important features of a conditional statement, e.g., the *depth* or *nesting* of a conditional within other conditionals. Sections 2.2 and 2.3 discussed two of the most obvious dimensions that are not respected by Definition 4: considerations of *incommensurability* and *order*, respectively.

Examination of Definition 4 reveals commitments to the following:

- $$t_{w}(\varphi )\oplus _{w}t_{w}(\psi )\le _{w}t_{w}(\varphi \rightarrow \psi )$$
- $$t_{w}(\varphi \rightarrow \psi )=t_{w}(\psi \rightarrow \varphi )$$.

The first condition shows the revised account to be inappropriate for modeling conditionals exhibiting the type of incommensurability we’d considered; the second shows the *t* of Definition 4 to be incapable of respecting intensional conditionals whose topics are sensitive to order.

Thus, although an improvement on the original topic-theoretic apparatus of $\textsf{PAI}$, tools to cover the full spectrum of potential topic-theoretic properties of intensional conditionals requires a deeper revision.

## Conditional-agnostic analytic implication

Now, let us consider a positive formalization of the core intuition. In this section, I will introduce a modification of Definition 1 that will induce a logic of *conditional-agnostic* $\textsf{PAI}$ (or $\textsf{CA}/\textsf{PAI}$) that is sufficiently flexible to not only allow *agnosticism* about properties considered in the foregoing but to allow the positive representation of strengthened conditions in case elements of that agnosticism should be resolved.

### Model theory of $\textsf{CA}/\textsf{PAI}$

First, I will introduce the structure of our model theory:

#### Definition 5

A $\textsf{CA}/\textsf{PAI}$ Fine model is a tuple $\langle W,R,\mathcal {T},\oplus ,\multimap ,v,{t},h\rangle$ differing from Definition 1 by the addition of:

- For each $w\in W$, $\multimap _{w}$ is a binary function from $\mathcal {T}_{w}\times \mathcal {T}_{w}\rightarrow \mathcal {T}_{w}$
- For all $w,w'$ such that $wRw'$, $h_{w,w'}:\mathcal {T}_{w}\rightarrow \mathcal {T}_{w'}$ is a homomorphism such that:
  - for atoms *p*, $$h_{w,w'}(t_{w}(p))=t_{w'}(p)$$
  - $$h_{w,w'}(a\oplus _{w}b)=h_{w,w'}(a)\oplus _{w'}h_{w,w'}(b)$$
  - $$h_{w,w'}(a\multimap _{w}b)=h_{w,w'}(a)\multimap _{w'}h_{w,w'}(b)$$.

The introduction of homomorphisms $h_{w,w'}$ acts as a generalization of the constraint in Definition 1 that topic inclusion between atoms is preserved across accessible worlds. Although in $\textsf{PAI}$ this condition ensures preservation of topic inclusion *in general*, general topic preservation in $\textsf{CA}/\textsf{PAI}$ requires these homomorphisms.

#### Lemma 1

If $$wRw'$$ then $$h_{w,w'}(t_{w}(\varphi ))=t_{w'}(\varphi )$$.

#### Proof

The basis step is established by definition, so suppose that the property holds of subformula of $\varphi$. In case $\varphi$ is a conjunction, we have:

$$\begin{aligned} h_{w,w'}(t_{w}(\psi \wedge \xi ))= & {} h_{w,w'}(t_{w}(\psi )\oplus _{w} t_{w}(\xi ))\\= & {} h_{w,w'}(t_{w}(\psi ))\oplus _{w'} h_{w,w'}(t_{w}(\xi ))\\= & {} t_{w'}(\psi )\oplus _{w'}t_{w'}(\xi )\\= & {} t_{w'}(\psi \wedge \xi ) \end{aligned}$$

Simple modifications yield arguments establishing the remaining connectives. $\square$

Lemma 1 leads to the persistence of topic inclusion in general.

#### Lemma 2

If $t_{w}(\varphi )\le _{w} t_{w}(\psi )$ and $wRw'$ then $t_{w'}(\varphi )\le _{w'} t_{w'}(\psi )$.

#### Proof

Suppose that $t_{w}(\varphi )\le _{w} t_{w}(\psi )$. This can be expanded to the identity $t_{w}(\varphi )\oplus _{w}t_{w}(\psi )=t_{w}(\psi )$. By applying $h_{w,w'}$ to both sides, we infer that $h_{w,w'}(t_{w}(\varphi )\oplus _{w}t_{w}(\psi ))=h_{w,w'}(t_{w}(\psi ))$ and, in turn, that $h_{w,w'}(t_{w}(\varphi ))\oplus _{w'}h_{w,w'}(t_{w}(\psi ))=h_{w,w'}(t_{w}(\psi ))$. By Lemma 1, this can be rewritten as $t_{w'}(\varphi )\oplus _{w'}t_{w'}(\psi )=t_{w'}(\psi )$, which can be shortened to $t_{w'}(\varphi )\le _{w'}t_{w'}(\psi )$ as needed. $\square$

I will retain the truth conditions from stock $\textsf{PAI}$ models, but update the clauses by which each ${t}_{w}$ assigns topics to formulae:

#### Definition 6

The topic assignment function ${t}_{w}$ is extended through the language:

- $${t}_{w}(\lnot \varphi )={t}_{w}(\varphi )$$
- $${t}_{w}(\varphi \star \psi )={t}_{w}(\varphi )\oplus _{w}{t}_{w}(\psi )$$ for $$\star $$ extensional
- $${t}_{w}(\varphi \rightarrow \psi )={t}_{w}(\varphi )\multimap _{w}{t}_{w}(\psi )$$.

Let us make a few comments about our $\multimap$ function.

First, as desired, there are virtually no assumptions about $a\multimap _{w}b$ besides its domain and range, which faithfully captures our agnosticism concerning ${t}_{w}(\varphi \rightarrow \psi )$. An immediate consequence is that the properties of Definition 4 that were criticized in the last section are resolved by Definition 5. *E.g.*, that ${t}_{w}(\varphi \rightarrow \psi )={t}_{w}(\psi \rightarrow \varphi )$ fails in virtue of the absence of a guarantor that $a\multimap _{w}b=b\multimap _{w}a$. The consequences are similarly clear in considering Parry’s axioms of Parry (1933). For example, the axiom [*A*11], i.e.,will fail because nothing is assumed concerning the relationship between $a\multimap _{w}b$ and $a\oplus _{w}b$.^6^

This is not to say that $\multimap _{w}$ is *hollow* and *inert*. Through its *functionality*, many validities are obtained for free. Noting that ${t}_{w}(\varphi \wedge \varphi )={t}_{w}(\varphi )$, that ${t}_{w}(\lnot \varphi \wedge \lnot \psi )={t}_{w}(\lnot (\varphi \vee \psi ))$, or that ${t}_{w}(\varphi \wedge (\psi \wedge \xi ))={t}_{w}((\varphi \wedge \psi )\wedge \xi )$ suffices to establish that the following validities:

- $$\vDash (\varphi \wedge \varphi )\rightarrow \varphi $$
- $$\vDash (\lnot \varphi \wedge \lnot \psi )\rightarrow \lnot (\varphi \vee \psi )$$
- $$\vDash (\varphi \wedge (\psi \wedge \xi ))\rightarrow ((\varphi \wedge \psi )\wedge \xi )$$

hold, respectively.

Finally, it is clear that stronger consequence relations can be defined by imposing additional semantic constraints on $\multimap _{w}$. One extreme witnesses this: By setting $\multimap _{w}=\oplus _{w}$, the model theory collapses to that of Definition 1.^7^ But, as we will see at the end of this section, myriad subtle extensions intermediate between $\textsf{CA}/\textsf{PAI}$ and $\textsf{PAI}$ can be defined by tightening the definition of $\multimap _{w}$.

### An axiom system for $\textsf{CA}/\textsf{PAI}$

Having introduced its model theory, we now turn to a modification of Parry’s axioms for $\textsf{PAI}$ to provide a Hilbert-style calculus for $\textsf{CA}/\textsf{PAI}$. I follow the following intuition in its formulation: Axioms of the form $\varphi \rightarrow \psi$ in which $\varphi$ or $\psi$ have $\rightarrow$ as the primary connective represent a thesis about *inclusion* of the topic of a conditional; a thesis about inclusion is only meaningful in the presence of the type of thesis about intensional conditionals’ topics that our agnosticism rules out. In such cases, we generally replace the main $\rightarrow$ operator of an axiom [*An*] with a  to yield an axiom $[An^{\dagger }]$. In other cases, I try to carry over as much of $\textsf{PAI}$ as possible.

Consequently, I follow Parry’s notation with $f(\varphi )$ is any formula in which $\varphi$ appears; treat $f(\psi )$ as the result of replacing one or more instances of $\varphi$ with $\psi$ in $f(\varphi )$. We have two further notational devices to consider. First, consider the following definition:

#### Definition 7

- $$\mathscr {L}_{\rightarrow }(p)=\lbrace p\rbrace $$
- $$\mathscr {L}_{\rightarrow }(\lnot \varphi )=\mathscr {L}_{\rightarrow }(\varphi )$$
- $$\mathscr {L}_{\rightarrow }(\varphi \mathrel {\star }\psi )=\mathscr {L}_{\rightarrow }(\varphi )\cup \mathscr {L}_{\rightarrow }(\psi )$$ for $$\star $$ extensional
- $$\mathscr {L}_{\rightarrow }(\varphi \rightarrow \psi )=\lbrace \langle \mathscr {L}_{\rightarrow }(\varphi ),\mathscr {L}_{\rightarrow }(\psi )\rangle \rbrace $$.

Then the formula $g(\varphi )$ is any formula such that $\mathscr {L}^{\rightarrow }(g(\varphi ))\supseteq \mathscr {L}^{\rightarrow }(\varphi )$. Finally, I use the notation $\textbf{t}_{\varphi }$ to represent the formula . This equips us to describe an axiomatization of $\textsf{CA}/\textsf{PAI}$:

#### Definition 8

The logic of *conditional agnostic analytic implication* $\textsf{CA}/\textsf{PAI}$ is determined by the following axioms:and rules:

Note that this $[{\textit{MOD}}]$ rule is required to grant modal strength to axioms like $[A13^{\dagger }]$. In stripping such axioms of their topic-theoretic inclusion, we had to remove their strict implication-like properties as well. $[{\textit{MOD}}]$ serves to grant axioms this force.

Despite its defensibility, the reflection of our agnosticism in the axioms undoubtedly impedes attempts to hew too closely to prior work on the topic, i.e., Dunn’s (1972), Urquhart’s (1973), Fine’s (1986), or Deutsch (1979). Many of the lemmas playing critical roles in these papers’ proofs rest on assumptions concerning the subject-matter of a conditional precluded by our agnosticism. *E.g.*, encoding necessity of $\varphi$ as $(\varphi \rightarrow \varphi )\rightarrow \varphi$ works in the case of $\textsf{PAI}$ or $\textsf{DAI}$ because a guarantee that ${t}(\varphi \rightarrow \varphi )={t}(\varphi )$; we not only *have* no such guarantee, but suspicion of its validity serves as a cornerstone of this effort.

It is worth noting that, just as Dunn shows for a related system in Dunn (1972), the following useful “normal form theorems” are demonstrable in our system. By taking instances of axioms or appealing to definitions, the following “normal forms” can be derived:

#### Lemma 3

The following are provable:

$$\begin{aligned} \begin{array}{ll} {[}T7] \quad \varphi \leftrightarrow \lnot \lnot \varphi &{}\\ {[}T8] \quad \varphi \wedge \varphi \leftrightarrow \varphi &{}\quad [T9]\quad \varphi \vee \varphi \leftrightarrow \varphi \\ {[}T10] \quad \varphi \wedge \psi \leftrightarrow \psi \wedge \varphi &{}\quad [T11]\quad \varphi \vee \psi \leftrightarrow \psi \vee \varphi \\ {[}T12] \quad \varphi \wedge (\psi \wedge \xi )\leftrightarrow (\varphi \wedge \psi )\wedge \xi &{}\quad [T13]\quad \varphi \vee (\psi \vee \xi )\leftrightarrow (\varphi \vee \psi )\vee \xi \\ {[}T14] \quad \varphi \wedge (\psi \vee \xi )\leftrightarrow (\varphi \wedge \psi )\vee (\varphi \wedge \xi ) &{}\quad [T15] \quad \varphi \vee (\psi \wedge \xi )\leftrightarrow (\varphi \vee \psi )\wedge (\varphi \vee \xi )\\ {[}T16] \quad \lnot (\varphi \wedge \psi )\leftrightarrow (\lnot \varphi \vee \lnot \psi ) &{}\quad [T17]\quad \lnot (\varphi \vee \psi )\leftrightarrow (\lnot \varphi \wedge \lnot \psi ) \\ \end{array} \end{aligned}$$

The formulae in Lemma 3 bear a striking resemblance to presentations of first-degree systems as equivalences such as Angell’s presentation of his $\textsf{AC}$ in Angell (1977). It is worth noting that for first-degree formulae $\varphi$, $\psi$ (i.e., those including no instances of analytic implication), $\varphi \leftrightarrow \psi$ will be provable precisely when $\varphi$ and $\psi$ are classically equivalent and share exactly the same variables.^8^

Soundness is easy to confirm by checking the axioms and rules against Definition 5. As [*D*2] is the lone axiom whose proof of validity is not entirely self-contained, we prove this:

#### Observation 2

Axiom [*D*2] is valid.

#### Proof

Suppose that $w\Vdash \varphi \rightarrow \psi$ and fix an arbitrary $w'\in w{\uparrow }$. The hypothesis ensures that if $w'\Vdash \varphi$ then $w'\Vdash \psi$ while Lemma 2 guarantees that $t_{w'}(\psi )\le _{w'}t_{w'}(\varphi )$ holds as well. Thus, $w'\Vdash \varphi \rightarrow \psi$. Thus, it vacuously holds that all accessible points $w'$ making true $\lnot (\varphi \rightarrow \psi )$ also make true $\varphi \rightarrow \psi$. By definition of $t_{w}$, it also follows that $t_{w}(\lnot (\varphi \rightarrow \psi ))=t_{w}(\varphi \rightarrow \psi )$. Having met the truth-theoretic and topic-theoretic conditions, we conclude that that $w\Vdash \lnot (\varphi \rightarrow \psi )\rightarrow (\varphi \rightarrow \psi )$, whence . $\square$

The validity of the remaining axioms is trivial, whence:

#### Theorem 1

The axioms and rules are sound with respect to the model theory.

Completeness, of course, is more tricky. We show completeness by appeal to the canonical model technique.

### The canonical model

We now consider the canonical model for $\textsf{CA}/\textsf{PAI}$. Several lemmas are saved for an appendix, but primary among them is the observation that the conditional of $\textsf{CA}/\textsf{PAI}$ satisfies a syntactic version of Routley’s “double-barreled analysis” described in Routley et al. (1982). Consider translations by which the *necessity* of $\varphi$ is captured by the formula $\textbf{t}_{\varphi }\rightarrow \varphi$ and inclusion of the subject-matter of $\psi$ in that of $\varphi$ is captured by $\textbf{t}_{\varphi }\rightarrow \textbf{t}_{\psi }$.

Then the syntactic reflection “double-barreled analysis” within a theory amounts to an equivalence between $\Gamma \vdash \varphi \rightarrow \psi$ and the joint conditions that  (i.e., $\rightarrow$ is an $\textsf{S4}$ strict conditional) and $\Gamma \vdash \textbf{t}_{\varphi }\rightarrow \textbf{t}_{\psi }$ (i.e., $\rightarrow$ requires subject-matter inclusion).

Lemmas 10, 12, and 15 (proven in the appendix) in concert permit us to provide the fundamental characterization of the $\rightarrow$ connective:

#### Theorem 2

$$\Gamma \vdash \varphi \rightarrow \psi $$ iff .

With Theorem 2 in hand, we can now begin to prove completeness by describing a canonical model. First, we consider several definitions. To introduce the canonical model’s accessibility relation, we provide the following definition:

#### Definition 9

Let $\Gamma$ be a theory. Then $\Gamma ^{\Box }=\lbrace \varphi \mid \textbf{t}_{\varphi }\rightarrow \varphi \in \Gamma \rbrace$.

As for the concept semilattice, we introduce several additional definitions:

#### Definition 10

For a maximally consistent theory $\Gamma$, define an equivalence relation $\sim _{\Gamma }$ such that $\varphi \mathrel {\sim _{\Gamma }}\psi$ if $\textbf{t}_{\varphi }\leftrightarrow \textbf{t}_{\psi }\in \Gamma$.

#### Definition 11

Let $\llbracket \varphi \rrbracket _{\Gamma }$ be the equivalence class of formulae induced by $\sim _{\Gamma }$. Define two functions $\oplus _{\Gamma }$ and $\multimap _{\Gamma }$

- $$\llbracket \varphi \rrbracket _{\Gamma }\oplus _{\Gamma }\llbracket \psi \rrbracket _{\Gamma }=\llbracket \varphi \wedge \psi \rrbracket _{\Gamma }$$
- $$\llbracket \varphi \rrbracket _{\Gamma }\multimap _{\Gamma }\llbracket \psi \rrbracket _{\Gamma }=\llbracket \varphi \rightarrow \psi \rrbracket _{\Gamma }$$

We will soon demonstrate that the above definitions satisfy appropriate properties. First, however, I will properly introduce the canonical model for a maximally consistent theory:

#### Definition 12

The $\textsf{CA}/\textsf{PAI}$ canonical model is $\mathfrak {M}=\langle W,R,\mathcal {T},\oplus ,\multimap ,v,{t},h\rangle$ where

- $$W=\lbrace \Delta \mid \Delta \text{ a } \text{ maximally } \text{ consistent } \text{ theory }\rbrace $$
- $$R=\lbrace \langle \Gamma ,\Xi \rangle \mid \Gamma ^{\Box }\subseteq \Xi \rbrace $$
- $$\Gamma \in v(p)$$ iff $$p\in \Gamma $$
- $${t}_{\Gamma }:\varphi \mapsto \llbracket \varphi \rrbracket _{\Gamma }$$ for each $$\Gamma \in W$$
- $$h_{\Gamma ,\Gamma '}(\llbracket \varphi \rrbracket _{\Gamma })=\llbracket \varphi \rrbracket _{\Gamma '}$$.

#### Definition 13

For $\Gamma$ a maximally consistent $\textsf{CA}/\textsf{PAI}$ theory, its canonical model $\mathfrak {M}_{\Gamma }$ is the submodel of $\mathfrak {M}$ where $W_{\Gamma }=\lbrace \Delta \mid \Gamma R\Delta \rbrace$.

Lemmas establishing that the above structures enjoy the appropriate properties to qualify as models are saved for the appendix, but I will state the penultimate step of the following fundamental lemma concerning the canonical model:

#### Lemma 4

$$\varphi \in \Gamma $$ iff $$\Gamma \Vdash \varphi $$.

Lemma 4, of course, immediately captures completeness of the axioms.

#### Theorem 3

The theorems of $\textsf{CA}/\textsf{PAI}$ are complete with respect to the model theory.

This definition guarantees that for every nontheorem of $\textsf{CA}/\textsf{PAI}$ a countermodel exists. As a consequence, we can employ the method of filtrations to establish the following theorem (a proof of which is found in the final appendix):

#### Theorem 4

The set of theorems of $\textsf{CA}/\textsf{PAI}$ is decidable.

Having benefited from the efficacy of the general definition of canonical model, we can appeal to small modifications in order to explore systems intermediate between $\textsf{CA}/\textsf{PAI}$ and $\textsf{PAI}$ (indeed, intermediate between $\textsf{CA}/\textsf{PAI}$ and Dunn’s $\textsf{DAI}$). The fine-grained distinctions that can be made in extensions of $\textsf{CA}/\textsf{PAI}$ provide an ample demonstration of the power of the proposal.

### Systems intermediate between $\textsf{CA}/\textsf{PAI}$ and $\textsf{PAI}$

The agnosticism at the heart of the foregoing analysis is not inflexible. In acknowledgment that some thesis or other about the subject-matter of $\varphi \rightarrow \psi$ may ultimately be found to be compelling, the framework allows one to impose tighter conditions on the model theory to generate stronger logics. Just as standard Kripke semantics allow one to align semantic constraints on *R* with characteristic axioms, so, too, may one align conditions on $\multimap _{w}$ with characteristic axioms, including many of those initially included by Parry.

In this section, we will examine a sampling of extensions of our $\textsf{CA}/\textsf{PAI}$ to illustrate how such constraints may be employed. Of course, we have observed $\textsf{PAI}$ itself may be generated by setting $\multimap _{w}=\oplus _{w}$. But we can examine extensions intermediate between these cases as well. Initially, we look at Parry’s axiom [*A*8].

$$\begin{aligned} \begin{array}{ll} [A8] &{}\quad (\varphi \rightarrow (\psi \wedge \xi ))\rightarrow (\varphi \rightarrow \psi )\\ \end{array} \end{aligned}$$

*N.b.* the intuitive difference between [*A*8] and $[A8^{\dagger }]$. $[A8^{\dagger }]$ says only that if $\varphi \rightarrow (\psi \wedge \xi )$ holds, then $\varphi \rightarrow \psi$ holds. Parry’s [*A*8] adds to this a topic-theoretic statement that the *topic* of $\varphi \rightarrow \psi$ is *included* in that of $\varphi \rightarrow (\psi \wedge \xi )$.

Of all of the $\textsf{PAI}$ axioms concerning topic inclusion between conditional sentences, [*A*8] seems to be the most compelling if one emphasizes the *analytic* component of analytic implication. Indeed, sentence **[3]** is essentially a reflection of this intuition. There is a strong sense in which e.g. the inference from “that Sam is a bachelor analytically implies that Sam is unmarried and Sam is a man” to “that Sam is a bachelor analytically implies that Sam is unmarried” is *itself* a sort of analysis of the meaning of the premise. The consequent arguably follows by some type of *decomposition* of the premise into its *parts*.

In keeping with Parry’s goal of a logic of *analytic implication*, there is a sense in which Axiom 8 communicates a true *analytic decomposition* of the subject-matter of $\varphi \rightarrow \psi \wedge \xi$. Taking this seriously, then, let us define a logic $\textsf{CA}/\textsf{PAI}_{8}$. I use $\oplus$ to indicate the inclusion of a new axiom.^9^ Then

#### Definition 14

$$\textsf{CA}/\textsf{PAI}_{8}=\textsf{CA}/\textsf{PAI}\oplus (\varphi \rightarrow (\psi \wedge \xi ))\rightarrow (\varphi \rightarrow \psi )$$.

Note that $\textsf{CA}/\textsf{PAI}_{8}$ is both an extension of $\textsf{CA}/\textsf{PAI}$ and a subsystem of $\textsf{PAI}$. We can ensure its validity against a particular class of $\textsf{CA}/\textsf{PAI}$ models by identifying an appropriate semantic condition on $\multimap$:

#### Definition 15

Call $\multimap _{w}$ *right decomposable* if the following condition holds:

- $$a\multimap _{w}b\le _{w}a\multimap _{w}(b\mathrel {\oplus }_{w}c)$$.

This allows us to state a theorem (proven in the appendix):

#### Theorem 5

$\textsf{CA}/\textsf{PAI}_{8}$ is characterized by models in which each $\multimap _{w}$ is right decomposable.

A similarly plausible strengthening might be to embrace Parry’s axiom [*A*7], that is:

$$\begin{aligned} \begin{array}{ll} [A7] &{}\quad ((\varphi \rightarrow \psi )\wedge (\psi \rightarrow \xi ))\rightarrow (\varphi \rightarrow \xi )\\ \end{array} \end{aligned}$$

While less obviously correct than [*A*8], there is nevertheless some clear intuitive appeal to [*A*7]. It admits an intuitive reading attesting to the *analyticity* of the hypothetical syllogism, according to which the inference introduces no subject-matter beyond that of its premises.

#### Definition 16

$$\textsf{CA}/\textsf{PAI}_{7}=\textsf{CA}/\textsf{PAI}\oplus ((\varphi \rightarrow \psi )\wedge (\psi \rightarrow \xi ))\rightarrow (\varphi \rightarrow \xi )$$.

We can provide a clear and elegant semantic constraint on $\multimap _{w}$ that will prove characteristic of this extension.

#### Definition 17

Call $\multimap _{w}$ *middle term eliminable* if the following condition holds:

- $$a\multimap _{w}c\le _{w}((a\multimap _{w}b)\mathrel {\oplus }_{w}(b\multimap _{w}c))$$.

Again, we can state the following (the proof of which is in the appendix):

#### Theorem 6

$\textsf{CA}/\textsf{PAI}_{7}$ is characterized by models in which each $\multimap _{w}$ is middle term eliminable.

The flexibility of the framework is reinforced by the ability to characterize myriad intermediate logics. Just as, e.g., $\textsf{S4}$ corresponds to the joint frame conditions of reflexivity and transitivity, we can jointly impart the above conditions to yield a stronger logic. Consider the following corollary:

#### Corollary 1

Let $\textsf{CA}/\textsf{PAI}_{7,8}=\textsf{CA}/\textsf{PAI}\oplus ((\varphi \rightarrow \psi )\wedge (\psi \rightarrow \xi ))\rightarrow (\varphi \rightarrow \xi )\oplus (\varphi \rightarrow (\psi \wedge \xi ))\rightarrow (\varphi \rightarrow \psi )$. Then $\textsf{CA}/\textsf{PAI}_{7,8}$ is characterized by models in which each $\multimap _{w}$ is both middle term eliminable and right decomposable.

This presents an opportunity to make good on a promissory note issued in Sect. 2.2 concerning the appropriate framework in case one rejects the possibility of *incommensurability*, i.e., insists on the preservation of subtopics in intensional cases. This preservation can be described as a semantic condition:

#### Definition 18

Call an $\textsf{CA}/\textsf{PAI}$ model *subtopic-preserving* if for all *w* and topics *a*, *b*, $a\oplus _{w}b\le _{w}a\multimap _{w}b$.

Interestingly, one of Parry’s axioms that had been used to illustrate the features of $\multimap$ in Sect. 4.1—[*A*11]—corresponds to this property. Following the foregoing idioms, we can define the following:

#### Definition 19

.

Then this extension axiomatizes the class of models in which this type of preservation is respected. We show this with the following characterization result (which will be proven in the appendices):

#### Theorem 7

$\textsf{CA}/\textsf{PAI}_{11}$ is characteristic of subtopic-preserving models.

Again, this speaks to the flexibility of the proposal as the framework is extensible enough to model differing positions about topic. This—and the other above examples—shows that we can in practice exercise a fine degree of control over the *topic-theoretic* features of models to determine systems intermediate between $\textsf{CA}/\textsf{PAI}$ and $\textsf{PAI}$ in the same way that imparting conditions to *R* yields stronger modal logics than $\textsf{K}$.

But the assignment of subject-matter is not the only dimension along which one might want to strengthen a logic. Recall, for example, Dunn’s *demodalized* $\textsf{DAI}$ that results from adding to $\textsf{PAI}$ the *demodalizer axiom* $\varphi \rightarrow (\lnot \varphi \rightarrow \varphi )$. Traditionally, the demodalizer axiom—which e.g. collapses $\textsf{S4}$ into classical logic—is considered to “collapse modal distinctions.” Such a collapse is reflected in $\textsf{DAI}$, which is essentially a “classical” logic of analytic implication (an assertion clear from the presentation by Epstein—who independently discovered $\textsf{DAI}$—cataloged in Epstein 1987).

In the present context, though, the stock demodalizer axiom collapses not only modal distinctions but collapses many topic-theoretic distinctions one may wish to preserve. The validity of $\varphi \rightarrow (\lnot \varphi \rightarrow \varphi )$ would require the imposition of new conditions on $\langle \mathcal {T}_{w},\oplus _{w}\rangle$, e.g., that $a\multimap _{w}a\le _{w}a$; this condition, of course, in in conflict with an intuition that intensional conditionals play a “transformative” role in the determination of their subject-matters.

So to provide a *strictly* demodalized $\textsf{CA}/\textsf{PAI}$—one lacking side-effects on the properties of the topic semilattices—requires a different axiom. We provide an alternative axiom by the following:

#### Definition 20

.

This suffices to characterize the logic (a proof can be found in the appendix):

#### Theorem 8

$\textsf{CA}/\textsf{DAI}$ is characterized by models in which *R* is the diagonal relation on *W*, i.e., $R=\lbrace \langle w,w\rangle \mid w\in W\rbrace$.

I also note that $\mathfrak {M}_{\Gamma }$ in the above case can be seen to be have a *singleton* domain *W*. Thus, soundness and completeness with respect to single-pointed frames is also established. Reading such models as ones in which the actual world is the *only* possible world, the necessity of a proposition is equivalent to its truth. Nevertheless, no additional conditions on $\multimap _{w}$ are induced. This further reinforces the notion that  effectively *demodalizes* the system without side-effects on its topic-theoretic machinery. Moreover, as the scheme $\textbf{t}_{\varphi }\rightarrow \varphi$ essentially provides a definition of necessity, it is worth pointing out that characteristic axioms for frame conditions for systems intermediate between $\textsf{S4}$ and $\textsf{Triv}$ can be emulated in the language of $\textsf{CA}/\textsf{PAI}$.

**Fig. 1:**
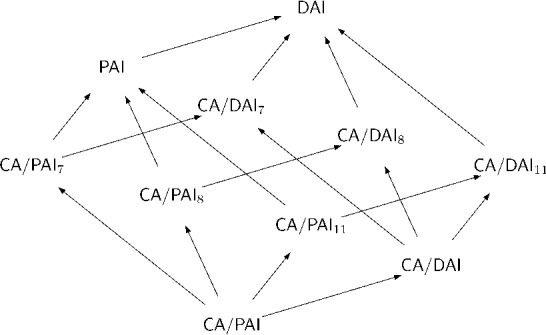
Logics Intermediate Between $\textsf{CA}/\textsf{PAI}$ and $\textsf{DAI}$

Consider extending the above naming conventions in the obvious ways (so that e.g. $\textsf{CA}/\textsf{DAI}_{7}$ extends $\textsf{CA}/\textsf{DAI}$ with axiom [*A*7]). Then between Theorems 5, 6, 7 and 8, a picture of logics intermediate between $\textsf{CA}/\textsf{PAI}$ and $\textsf{DAI}$ emerges, pictured in Fig. 1.

Given that one can express necessity, further intermediate logics can be determined by including axioms cognate with the standard axioms or imposing characteristic frame conditions on *R*. Fine’s remarks e.g. on axiomatizing $\textsf{S5}$-like extensions of Parry’s $\textsf{PAI}$ in Fine (1986) can be easily carried over to the case of $\textsf{CA}/\textsf{PAI}$. Given appropriate translations of boxes and diamonds, canonical model proofs for extensions of $\textsf{S4}$ will apply immediately to extensions of $\textsf{CA}/\textsf{PAI}$.

## Concluding remarks

In this paper, I have introduced machinery to allow fine-grained control over the subject-matter of an intensional conditional and have provided an initial implementation—a *proof of concept*, so to speak—by enriching Kit Fine’s model theory for $\textsf{PAI}$ and providing sound and complete axiomatizations of several logics in this neighborhood.

The reader is reminded that Definition 5 can be straightforwardly adapted to apply to cases beyond those envisioned by Parry. In particular, the model theory could be modified to allow for *other* intensional conditionals beyond Parry’s analytic implication. The reader should be able to intuit how additional accessibility relations added to the model (e.g., a universal accessibility relation $R'$ supporting the evaluation of an $\textsf{S5}$ strict implication ) can be harmonized with corresponding functions defined analogously to $\multimap$.

*E.g.*, complementing the function $\multimap$ with a function  would permit the representation of precise relationships between the subject-matters of $\varphi \rightarrow \psi$ and . By further augmentation to the model theory, a plethora of conditionals can receive their own $\multimap$ function.

Of course, this opens the doors to theories of topic for intensional notions *beyond* conditionals. Because one can define e.g. $\textsf{S4}$ necessity in virtue of a strict conditional, accounts of the topics for unary modal operators of all sorts are within the scope of the current proposal. Likewise, as variably strict conditionals themselves, the analysis of topics of *TSIMs themselves* is available merely by adding new $\multimap$ functions for each TSIM.

The present paper is intended as the first in a sequence of several detailed examinations of topic-theoretic properties of intensional connectives and operators. In the planned sequel, the theory is extended to provide accounts of topic for *intensional conditionals in general* and *unary modal operators*. These accounts will be directly embedded in the setting of TSIMs, which will provide an account of the topic of formulae in which TSIMs appear.

## Appendix A: The double-barreled analysis

This section includes proofs of lemmas necessary to prove Theorem 2. Initially, we observe some straightforward properties.

### Lemma 5

$$\vdash \varphi \rightarrow \varphi $$.

### Lemma 6

The following are provable:

### Proof

These are recognizably instances of $[A13^{\dagger }]$ in case $g(\varphi )$ is $\varphi$ itself, $\varphi \vee \lnot \varphi \vee \psi$, and $\varphi \vee \lnot \varphi \vee \psi \vee \xi$, respectively. $\square$

We will proceed now to demonstrating that such an equivalence holds.

### Lemma 7

If $$\Gamma \vdash \varphi \rightarrow \psi $$ then $$\Gamma \vdash \textbf{t}_{\varphi \rightarrow \psi }\rightarrow (\varphi \rightarrow \psi )$$.

### Proof

Suppose that $\Gamma \vdash \varphi \rightarrow \psi$. By [*D*2] and [*MP*], $\Gamma \vdash \lnot (\varphi \rightarrow \psi )\rightarrow (\varphi \rightarrow \psi )$. By Lemma 5, also $\Gamma \vdash (\varphi \rightarrow \psi )\rightarrow (\varphi \rightarrow \psi )$. So by $[A10^{\dagger }]$ and [*T*9], we conclude that $\Gamma \vdash (\lnot (\varphi \rightarrow \psi )\vee (\varphi \rightarrow \psi ))\rightarrow (\varphi \rightarrow \psi )$, i.e., $\Gamma \vdash \textbf{t}_{\varphi \rightarrow \psi }\rightarrow (\varphi \rightarrow \psi )$. $\square$

### Lemma 8

$$\vdash (\textbf{t}_{\varphi }\wedge \textbf{t}_{\psi })\leftrightarrow \textbf{t}_{\varphi \wedge \psi }$$.

### Proof

For left-to-right, note that by some permutations and definitions, we can establish that $\vdash (\textbf{t}_{\varphi }\wedge \textbf{t}_{\psi })\leftrightarrow [[(\varphi \wedge \psi )]\vee [(\lnot \varphi \wedge \psi )\vee (\varphi \wedge \lnot \psi )\vee (\lnot \varphi \wedge \lnot \psi )]]$. First, by Lemma 5, $\vdash (\varphi \wedge \psi )\rightarrow (\varphi \wedge \psi )$. Second, straightforward appeals to $[A10^{\dagger }]$, [*T*9], and [*T*16] show that $\vdash [(\lnot \varphi \wedge \psi )\vee (\varphi \wedge \lnot \psi )\vee (\lnot \varphi \wedge \lnot \psi )]\rightarrow \lnot (\varphi \wedge \psi )$. Thus, $\vdash [[(\varphi \wedge \psi )]\vee [(\lnot \varphi \wedge \psi )\vee (\varphi \wedge \lnot \psi )\vee (\lnot \varphi \wedge \lnot \psi )]]\rightarrow [\lnot (\varphi \wedge \psi )\vee (\varphi \wedge \psi )]$. Recognizing the consequent of this formula as $\textbf{t}_{\varphi \wedge \psi }$, appeal to $[A7^{\dagger }]$ establishes that $\vdash (\textbf{t}_{\varphi }\wedge \textbf{t}_{\psi })\rightarrow \textbf{t}_{\varphi \wedge \psi }$.

For right-to-left, it is straightforward to show that $\vdash \textbf{t}_{\varphi \wedge \psi }\rightarrow [(\varphi \vee \lnot \varphi \vee \lnot \psi )\wedge (\psi \vee \lnot \psi \vee \lnot \varphi )]$. By Lemma 6, both $\vdash (\varphi \vee \lnot \varphi \vee \lnot \psi )\rightarrow \textbf{t}_{\varphi }$ and $\vdash (\psi \vee \lnot \psi \vee \lnot \varphi )\rightarrow \textbf{t}_{\psi }$, whence appeals to $[A7^{\dagger }]$ and $[A9^{\dagger }]$ establish that $\vdash \textbf{t}_{\varphi \wedge \psi }\rightarrow (\textbf{t}_{\varphi }\wedge \textbf{t}_{\psi })$. $\square$

### Lemma 9

If $$\Gamma \vdash \varphi \rightarrow \psi $$ then $$\Gamma \vdash \varphi \leftrightarrow (\varphi \wedge \psi )$$.

### Proof

Applying $[A8^{\dagger }]$ to the identity $(\varphi \wedge \psi )\rightarrow (\varphi \wedge \psi )$ yields $(\varphi \wedge \psi )\rightarrow \varphi$. For the other direction, an application of $[A9^{\dagger }]$ , so by [*ADJ*] and hypothetical syllogism, we easily infer $\varphi \rightarrow (\varphi \wedge \psi )$. $\square$

The above lemmas permit us to demonstrate that the $\rightarrow$ connective continues to satisfy the requisite property that provability of $\varphi \rightarrow \psi$ implies the *inclusion of subject-matter*:

### Lemma 10

If $$\Gamma \vdash \varphi \rightarrow \psi $$ then $$\Gamma \vdash \textbf{t}_{\varphi }\rightarrow \textbf{t}_{\psi }$$.

### Proof

Suppose that $\Gamma \vdash \varphi \rightarrow \psi$. Then by Lemma 9, we have $\Gamma \vdash \varphi \leftrightarrow (\varphi \wedge \psi )$ and by Lemma 5 we have . By [*ADJ*], we can conjoin these and use an instance of $[A12^{\dagger }]$ to yield . By definitions, instances of distribution, and applications of $[A7^{\dagger }]$, we can infer that ; by $[A8^{\dagger }]$, .

But $(\psi \vee \lnot \psi \vee \lnot \varphi )\rightarrow (\psi \vee \lnot \psi )$ is a theorem by Lemma 6, and by applying $[A7^{\dagger }]$, we infer that , i.e., $\textbf{t}_{\varphi }\rightarrow \textbf{t}_{\psi }$. $\square$

The second component of the analysis of the conditional can now be demonstrated, namely, that the conditional $\rightarrow$ acts as a strict $\textsf{S4}$ conditional. We require a simple lemma first:

### Lemma 11

.

### Proof

By definitions and applications of distribution, we infer theoremhood of  and, by $[A8^{\dagger }]$, . By Lemma 6, we infer that $(\varphi \vee \lnot \varphi \vee \psi )\rightarrow (\varphi \vee \lnot \varphi )$, and by $[A7^{\dagger }]$, , i.e., . $\square$

Lemma 11 allows us to show that validity of $\varphi \rightarrow \psi$ has properties like that of a strict conditional:

### Lemma 12

If $$\Gamma \vdash \varphi \rightarrow \psi $$ then .

### Proof

By Lemma 5 and hypothesis, [*ADJ*] yields $\Gamma \vdash (\varphi \rightarrow \psi )\wedge (\lnot \varphi \rightarrow \lnot \varphi )$; by an instance of $[A10^{\dagger }]$, we thereby may infer that $\Gamma \vdash (\varphi \vee \lnot \varphi )\rightarrow (\lnot \varphi \vee \psi )$, i.e., . By an appropriate instance of $[A9^{\dagger }]$, we appeal to Lemma 11 to infer that . $\square$

### Lemma 13

If  then $$\Gamma \vdash \varphi \rightarrow \psi $$.

### Proof

By Lemma 5, the hypothesis, and [*ADJ*], $\Gamma \vdash (\varphi \rightarrow \varphi )\wedge (\varphi \rightarrow (\lnot \varphi \vee \psi ))$. By appeal to appropriate instances of [*A*2] and $[A9^{\dagger }]$, we infer that $\Gamma \vdash \varphi \rightarrow (\varphi \wedge (\lnot \varphi \vee \psi ))$ and, by [*A*5], $\Gamma \vdash \varphi \rightarrow ((\varphi \wedge \lnot \varphi )\vee (\varphi \wedge \psi ))$. By an instance of [*A*6], we continue to infer that $\Gamma \vdash \varphi \rightarrow (\varphi \wedge \psi )$ and by theoremhood of $(\varphi \wedge \psi )\rightarrow \psi$, [*ADJ*] and $[A7^{\dagger }]$ give us $\Gamma \vdash \varphi \rightarrow \psi$. $\square$

### Lemma 14

If $$\Gamma \vdash \textbf{t}_{\varphi }\rightarrow \textbf{t}_{\psi }$$ then .

### Proof

Suppose that $\Gamma \vdash \textbf{t}_{\varphi }\rightarrow \textbf{t}_{\psi }$, i.e., $\Gamma \vdash (\varphi \vee \lnot \varphi )\rightarrow (\psi \vee \lnot \psi )$. Then by Lemma 5, $(\varphi \vee \lnot \varphi )\rightarrow (\varphi \vee \lnot \varphi )$ is a theorem, and an appeal to $[A10^{\dagger }]$ ensures that $\Gamma \vdash (\varphi \vee \lnot \varphi )\rightarrow (\varphi \vee \lnot \varphi \vee \psi \vee \lnot \psi )$.

Now, by Lemma 6, $(\varphi \vee \lnot \varphi \vee \psi \vee \lnot \psi )\rightarrow (\varphi \vee \lnot \varphi \vee \psi )$ and $(\varphi \vee \psi \vee \lnot \psi )\rightarrow (\varphi \vee \lnot \varphi \vee \psi \vee \lnot \psi )$ are also theorems. Between [*A*2] and $[A9^{\dagger }]$, then, we can infer that $(\varphi \vee \lnot \varphi )\rightarrow ((\varphi \vee \lnot \varphi \vee \psi )\wedge (\varphi \vee \lnot \varphi \vee \psi \vee \lnot \psi ))$. But because, as we saw in earlier lemmas, distributions and definitions ensure theoremhood of , $[A9^{\dagger }]$ ensures that , i.e., . $\square$

The foregoing lemmas allow us to show that the conjunction of appropriate sentential representations of topic inclusion and strict conditional behavior are *sufficient* to prove the corresponding conditional $\varphi \rightarrow \psi$:

### Lemma 15

If $\Gamma \vdash \textbf{t}_{\varphi }\rightarrow \textbf{t}_{\psi }$ and  then $\Gamma \vdash \varphi \rightarrow \psi$.

### Proof

Suppose that $\Gamma \vdash \textbf{t}_{\varphi }\rightarrow \textbf{t}_{\psi }$. Then by Lemma 14, . By Lemma 6, $\varphi \rightarrow \textbf{t}_{\varphi }$, whence $[A7^{\dagger }]$ guarantees that . Applying $[A7^{\dagger }]$ again to the hypothesis that  in turn yields that . But by Lemma 13, this entails that $\Gamma \vdash \varphi \rightarrow \psi$. $\square$

## Appendix B: Properties of the canonical model

This section includes lemmas necessary to establish that the canonical model is well-defined. First, we show that topic assignments persist along *R* as required:

### Observation 3

If $\Gamma R\Delta$ and $\varphi \le _{\Gamma }\psi$ then $\varphi \le _{\Delta }\psi$.

### Proof

The hypothesis that $\varphi \le _{\Gamma }\psi$ means that $\textbf{t}_{\psi }\rightarrow \textbf{t}_{\varphi }\in \Gamma$. By Lemma 7, $\Gamma \vdash \textbf{t}_{\textbf{t}_{\psi }\rightarrow \textbf{t}_{\varphi }}\rightarrow (\textbf{t}_{\psi }\rightarrow \textbf{t}_{\varphi })$, whence $\textbf{t}_{\psi }\rightarrow \textbf{t}_{\varphi }\in \Gamma ^{\Box }$. The hypothesis that $\Gamma R\Delta$ means that $\Gamma ^{\Box }\subseteq \Delta$, whence $\textbf{t}_{\psi }\rightarrow \textbf{t}_{\varphi }\in \Delta$ and $\varphi \le _{\Delta }\psi$. $\square$

We show that *R* has the necessary properties so that $\langle W,R\rangle$ is an $\textsf{S4}$ Kripke frame:

### Observation 4

*R* is reflexive and transitive.

### Proof

First, we examine reflexivity. For any $\varphi \in \Gamma ^{\Box }$, $\textbf{t}_{\varphi }\rightarrow \varphi \in \Gamma$. But as a tautology, $\textbf{t}_{\varphi }\in \Gamma$ and by [*MP*], $\varphi \in \Gamma$. Thus, $\Gamma ^{\Box }\subseteq \Gamma$, i.e., $\Gamma R\Gamma$.

Second, we show transitivity. Suppose that $\Gamma R\Xi R\Theta$. We show $\Gamma R\Theta$. Pick a $\varphi \in \Gamma ^{\Box }$. Then $\textbf{t}_{\varphi }\rightarrow \varphi \in \Gamma$. By Lemma 7, also $\textbf{t}_{\textbf{t}_{\varphi }\rightarrow \varphi }\rightarrow (\textbf{t}_{\varphi }\rightarrow \varphi )\in \Gamma$. Thus, $\textbf{t}_{\varphi }\rightarrow \varphi$ is an element of $\Gamma ^{\Box }$, and thus an element of $\Xi$. But if $\textbf{t}_{\varphi }\rightarrow \varphi \in \Xi$, $\varphi \in \Xi ^{\Box }$, whence $\varphi \in \Theta$. $\square$

We finally need to demonstrate that our definitions for $\oplus$, $\multimap$, and homomorphisms *h* are well-defined, that is, that these are in fact functions. We start with $\oplus _{\Gamma }$:

### Lemma 16

Each $\oplus _{\Gamma }$ is a function.

### Proof

Without loss of generality, that $\oplus _{\Gamma }$ is a function means that if $\textbf{t}_{\varphi }\leftrightarrow \textbf{t}_{\psi }\in \Gamma$ then $\textbf{t}_{\varphi \wedge \xi }\leftrightarrow \textbf{t}_{\psi \wedge \xi }\in \Gamma$. Assume that the antecedent condition holds; then by selecting instances of $[A9^{\dagger }]$ and the theorem $\textbf{t}_{\xi }\rightarrow \textbf{t}_{\xi }$, we may straightforwardly infer that $(\textbf{t}_{\varphi }\wedge \textbf{t}_{\xi })\leftrightarrow (\textbf{t}_{\psi }\wedge \textbf{t}_{\xi })\in \Gamma$. By Lemma 8, we have as theorems $\textbf{t}_{\varphi \wedge \xi }\leftrightarrow (\textbf{t}_{\varphi }\wedge \textbf{t}_{\xi })$ and $\textbf{t}_{\psi \wedge \xi }\leftrightarrow (\textbf{t}_{\psi }\wedge \textbf{t}_{\xi })$. Thus, by several applications of rules to appropriate instances of $[A7^{\dagger }]$, we can infer that $\textbf{t}_{\varphi \wedge \xi }\leftrightarrow \textbf{t}_{\psi \wedge \xi }\in \Gamma$. So in case $\llbracket \varphi \rrbracket _{\Gamma }=\llbracket \varphi '\rrbracket _{\Gamma }$ and $\llbracket \psi \rrbracket _{\Gamma }=\llbracket \psi '\rrbracket _{\Gamma }$, we are guaranteed that $\llbracket \varphi \rrbracket _{\Gamma }\oplus _{\Gamma }\llbracket \psi \rrbracket _{\Gamma }=\llbracket \varphi '\rrbracket _{\Gamma }\oplus _{\Gamma }\llbracket \psi '\rrbracket _{\Gamma }$. $\square$

We continue by showing $\multimap _{\Gamma }$ is well-defined as well:

### Lemma 17

Each $\multimap _{\Gamma }$ is a function.

### Proof

Suppose that $\textbf{t}_{\varphi }\leftrightarrow \textbf{t}_{\psi }\in \Gamma$. Then as a consequence of [*D*1], both $\textbf{t}_{\varphi \rightarrow \xi }\leftrightarrow \textbf{t}_{\psi \rightarrow \xi }\in \Gamma$ and $\textbf{t}_{\xi \rightarrow \varphi }\leftrightarrow \textbf{t}_{\xi \rightarrow \psi }\in \Gamma$. Thus, if $\llbracket \varphi \rrbracket _{\Gamma }=\llbracket \varphi '\rrbracket _{\Gamma }$ and $\llbracket \psi \rrbracket _{\Gamma }=\llbracket \psi '\rrbracket _{\Gamma }$, $\llbracket \varphi \rrbracket _{\Gamma }\multimap _{\Gamma }\llbracket \psi \rrbracket _{\Gamma }=\llbracket \varphi '\rrbracket _{\Gamma }\multimap _{\Gamma }\llbracket \psi '\rrbracket _{\Gamma }$. $\square$

We conclude our survey of the canonical model’s functions by establishing that the functions $h_{\Gamma ,\Gamma '}$ are indeed homomorphisms.

### Lemma 18

Each $h_{\Gamma ,\Gamma '}$ is a homomorphism.

### Proof

Glancing at Definition 12 suffices to establishes the functionality of each $h_{\Gamma ,\Gamma '}$ as well as the property that $h_{\Gamma ,\Gamma '}(t_{\Gamma }(p))=t_{\Gamma '}(p)$ for atoms *p*. Thus, we turn the preservation of structure. In the case of fusion, the following equivalences hold:

$$\begin{aligned} h_{\Gamma ,\Gamma '}(\llbracket \varphi \rrbracket _{\Gamma }\oplus _{\Gamma }\llbracket \psi \rrbracket _{\Gamma })&= h_{\Gamma ,\Gamma '}(\llbracket \varphi \wedge \psi \rrbracket _{\Gamma })&\text {Lemma}\;16\\&= \llbracket \varphi \wedge \psi \rrbracket _{\Gamma '}&\text {Definition}\;12\\&= \llbracket \varphi \rrbracket _{\Gamma '}\oplus _{\Gamma '}\llbracket \psi \rrbracket _{\Gamma '}&\text {Lemma}\;16\\&= h_{\Gamma ,\Gamma '}(\llbracket \varphi \rrbracket _{\Gamma })\oplus _{\Gamma '}h_{\Gamma ,\Gamma '}(\llbracket \psi \rrbracket _{\Gamma })&\text {Definition}\;12\\ \end{aligned}$$

The case of $\multimap$ follows from a modification of the above chain of identities by exchanging function symbols and connectives and appealing to Lemma 17 as appropriate. $\square$

Having shown that *R* and the concept lattices have the appropriate properties, we conclude that $\mathfrak {M}$ (and each $\mathfrak {M}_{\Gamma }$) is in fact a $\textsf{CA}/\textsf{PAI}$ model.

For convenience, we provide the following observation:

### Observation 5

$$\llbracket \varphi \rrbracket _{\Gamma }\le _{\Gamma }\llbracket \psi \rrbracket _{\Gamma }$$ iff $$\textbf{t}_{\psi }\rightarrow \textbf{t}_{\varphi }\in \Gamma $$.

### Proof

Because $a\le _{\Gamma }b$ is shorthand for $b=a\oplus _{\Gamma } b$, the requirement is to prove that $\Gamma \vdash \textbf{t}_{\psi \wedge \varphi }\leftrightarrow \textbf{t}_{\psi }$ iff $\Gamma \vdash \textbf{t}_{\psi }\rightarrow \textbf{t}_{\varphi }$. But because $\vdash \textbf{t}_{\psi \wedge \varphi }\rightarrow \textbf{t}_{\psi }$ can be easily established, the requirement is simply to show that $\Gamma \vdash \textbf{t}_{\psi }\rightarrow \textbf{t}_{\psi \wedge \varphi }$ iff $\Gamma \vdash \textbf{t}_{\psi }\rightarrow \textbf{t}_{\varphi }$.

For left-to-right, suppose that $\Gamma \vdash \textbf{t}_{\psi }\rightarrow \textbf{t}_{\psi \wedge \varphi }$. Then appeal to Lemma 8 and $[A8^{\dagger }]$ assures us that $\textbf{t}_{\psi \wedge \varphi }\rightarrow \textbf{t}_{\varphi }$; by $[A7^{\dagger }]$, then $\Gamma \vdash \textbf{t}_{\psi }\rightarrow \textbf{t}_{\varphi }$.

For right-to-left, suppose that $\Gamma \vdash \textbf{t}_{\psi }\rightarrow \textbf{t}_{\varphi }$. By e.g. Lemma 5 and $[A9^{\dagger }]$, $\Gamma \vdash \textbf{t}_{\psi }\rightarrow (\textbf{t}_{\psi }\wedge \textbf{t}_{\varphi })$; but Lemma 8 again establishes equivalence between the consequent and $\textbf{t}_{\psi \wedge \varphi }$ itself, whence $\Gamma \vdash \textbf{t}_{\psi }\rightarrow \textbf{t}_{\psi \wedge \varphi }$. $\square$

Before proving Lemma 4 we prove important *closure properties* holding of $\Gamma ^{\Box }$.

### Lemma 19

For $\varphi$ a theorem of $\textsf{S}/\textsf{PAI}$, $\varphi \in \Gamma ^{\Box }$.

### Proof

We prove this by induction on complexity of proofs. As a basis step when $\varphi$ is an axiom:

- By Lemma 5, $\varphi \rightarrow \varphi \in \Gamma$ and by rule $[{\textit{MOD}}]$ $\lnot \varphi \rightarrow \varphi \in \Gamma$. Between $[A10^{\dagger }]$ and [*T*9], we can infer that $\textbf{t}_{\varphi }\rightarrow \varphi \in \Gamma$, whence $\varphi \in \Gamma ^{\Box }$.

Otherwise, $\varphi$ has been proven by applying one of the four rules to other sentences for which the property has been demonstrated. In order:

- For [*ADJ*] where $\varphi =\psi \wedge \psi$, the assumption is that $\Gamma$ includes $\textbf{t}_{\psi }\rightarrow \xi$ and $\textbf{t}_{\xi }\rightarrow \xi$. Through the use of $[A9^{\dagger }]$, we can infer that $\Gamma$ includes $(\textbf{t}_{\varphi }\wedge \textbf{t}_{\psi })\rightarrow \varphi \wedge \psi$ and by Lemma 8, ultimately infer that $\textbf{t}_{\varphi \wedge \psi }\rightarrow \varphi \wedge \psi \in \Gamma$, whence $\varphi \wedge \psi \in \Gamma ^{\Box }$.
- For [*MP*1], suppose that  and $\psi \in \Gamma ^{\Box }$. Then  and $\textbf{t}_{\psi }\rightarrow \psi \in \Gamma$. By an appeal to $[A9^{\dagger }]$, we can infer that $\Gamma$ includes . By appeal to Lemma 8, we infer the inclusion of  and—with a bit of permutation—an appeal to [*A*6] will get . Putting these together, we get
  Of course, it is provable that , whence by an appeal to an appropriate instance of $[A14^{\dagger }]$, we are able to conclude that $\lnot \varphi \rightarrow \varphi \in \Gamma$. Because $\varphi \rightarrow \varphi \in \Gamma$, it nearly immediately follows that $\textbf{t}_{\varphi }\rightarrow \varphi$, i.e., that $\varphi \in \Gamma ^{\Box }$.
- For [*MP*2], suppose that $\psi \rightarrow \varphi \in \Gamma ^{\Box }$. Then by the basis step of the inclusion of axioms we infer that $\Gamma ^{\Box }$ includes an appropriate copy of $[A11^{\dagger }]$ from which an appeal to the earlier proof of closure under [*MP*1] establishes that . Then if $\psi \in \Gamma ^{\Box }$ then $\varphi \in \Gamma ^{\Box }$ follows from these same reasons.
- An application of $[{\textit{MOD}}]$ entails that $\lnot \varphi \rightarrow \varphi \in \Gamma$ as it is an axiom. An appeal to our earlier Lemma 7 ensures that $\Gamma$ also includes $\textbf{t}_{\lnot \varphi \rightarrow \varphi }\rightarrow (\lnot \varphi \rightarrow \varphi )$, i.e., that $\lnot \varphi \rightarrow \varphi \in \Gamma ^{\Box }$. $\square$

The foregoing permit us to infer a critical lemma and corollary that illuminate the workings of elements of $\Gamma ^{\Box }$ as regards the theories extending it.

### Lemma 20

$\Gamma ^{\Box }\vdash \varphi$ iff for all $\Xi \supseteq \Gamma ^{\Box }$, $\varphi \in \Xi$.

### Proof

Left-to-right holds by definition. Suppose that $\varphi$ is a member of every theory $\Xi \supseteq \Gamma ^{\Box }$. Then $\Gamma ^{\Box }\vdash \varphi$ (for otherwise, simple reasoning would show the existence of an extension in which $\lnot \varphi$ holds). By the compactness of $\textsf{S}/\textsf{PAI}$, there exists a sequence of formulae $\xi _{0},\ldots ,\xi _{n-1}\in \Gamma ^{\Box }$ such that $\bigwedge _{i<n}\xi _{i}\vdash \varphi$. Importantly, by closure under [*ADJ*], $\bigwedge _{i<n}\xi _{i}\in \Gamma ^{\Box }$. By the classical deduction theorem,  is an $\textsf{S}/\textsf{PAI}$ theorem, whence by closure under [*ADJ*], . As $\bigwedge _{i<n}\xi _{i}\in \Gamma ^{\Box }$, by closure under [*MP*1] $\varphi \in \Gamma ^{\Box }$. $\square$

Importantly, this allows us to show that the syntactic behavior of  in $\Gamma ^{\Box }$ behaves like an $\textsf{S4}$ strict conditional:

### Corollary 2

iff for all $\Xi \supseteq \Gamma ^{\Box }$ such that $\varphi \in \Xi$, also $\psi \in \Xi$.

This brings us to the proof of Lemma 4:

### Proof

In the atomic case, this is handled by the valuation function *v*. Thus, as induction hypothesis, suppose that the result has been established for all formulae of lesser complexity than $\varphi$

- In case $\varphi =\lnot \psi$, $\lnot \psi \in \Gamma$ holds if (by maximality of $\Gamma$) and only if (by consistency of $\Gamma$) $\psi \notin \Gamma$. But by induction hypothesis, this is equivalent to $\Gamma \nVdash \psi$, i.e., $\Gamma \Vdash \lnot \psi$.
- In case $\varphi = \psi \wedge \xi$, $\psi \wedge \xi \in \Gamma$ if (by [*ADJ*]) and only if (by theoremhood of $(\psi \wedge \xi )\rightarrow \psi$ and $(\psi \wedge \xi )\rightarrow \xi$) both $\psi \in \Gamma$ and $\xi \in \Gamma$. By induction hypothesis, this is equivalent to the conjunction of $\Gamma \Vdash \psi$ and $\Gamma \Vdash \xi$, a condition which is equivalent to $\Gamma \Vdash \psi \wedge \xi$.
- Finally, consider the case in which $\varphi =\psi \rightarrow \xi$. By Theorem 2, $\psi \rightarrow \xi \in \Gamma$ holds precisely when  and $\textbf{t}_{\psi }\rightarrow \textbf{t}_{\xi }\in \Gamma$.
- holds exactly when , i.e., when at every $\Xi$ such that $\Gamma R\Xi$, if $\psi \in \Xi$ then $\xi \in \Xi$. By induction hypothesis and Corollary 2, this is equivalent to saying that at all accessible $\Xi$, if $\Xi \Vdash \psi$ then $\Xi \Vdash \xi$
- $\textbf{t}_{\psi }\rightarrow \textbf{t}_{\xi }\in \Gamma$ holds if and only if $\psi \wedge \xi \mathrel {\sim }_{\Gamma }\psi$, i.e., if $\llbracket \psi \rrbracket _{\Gamma }=\llbracket \psi \rrbracket _{\Gamma }\oplus _{\Gamma }\llbracket \xi \rrbracket _{\Gamma }$. But by construction, this is equivalent to ${t}_{\Gamma }(\xi )\le _{\Gamma }{t}_{\Gamma }(\psi )$ by Observation 5.

Jointly, the two above semantic conditions are equivalent to $\Gamma \Vdash \psi \rightarrow \xi$. $\square$

## Appendix C: Extensions of $\textsf{CA}/\textsf{PAI}$

In this section, we include proofs of various theorems characterizing extensions of $\textsf{CA}/\textsf{PAI}$. First is the proof of Theorem 5 characterizing the extension $\textsf{CA}/\textsf{PAI}_{8}$:

### Proof

Soundness is easily confirmed, so we consider completeness. Let $\Gamma$ be a maximally consistent $\textsf{CA}/\textsf{PAI}\oplus (\varphi \rightarrow (\psi \wedge \xi ))\rightarrow (\varphi \rightarrow \psi )$ theory. Then consider three arbitrary points from $\mathcal {T}_{\Gamma }$ $\llbracket \varphi \rrbracket _{\Gamma }$, $\llbracket \psi \rrbracket _{\Gamma }$ and $\llbracket \xi \rrbracket _{\Gamma }$. By Lemma 10, the hypothesis that $\Gamma \vdash (\varphi \rightarrow (\psi \wedge \xi ))\rightarrow (\varphi \rightarrow \psi )$ entails that $\Gamma \vdash \textbf{t}_{\varphi \rightarrow (\psi \wedge \xi )}\rightarrow \textbf{t}_{\varphi \rightarrow \psi }$. So

$$\begin{aligned} \llbracket \varphi \rrbracket _{\Gamma }\multimap _{\Gamma }\llbracket \psi \rrbracket _{\Gamma }=\llbracket \varphi \rightarrow \psi \rrbracket _{\Gamma }\le _{\Gamma }\llbracket \varphi \rightarrow (\psi \wedge \xi )\rrbracket _{\Gamma }=\llbracket \varphi \rrbracket _{\Gamma }\multimap _{\Gamma }(\llbracket \psi \rrbracket _{\Gamma }\oplus _{\Gamma }\llbracket \xi \rrbracket _{\Gamma }) \end{aligned}$$

Since the points in $\mathcal {T}_{\Gamma }$ were chosen arbitrarily, the property holds of any $\textsf{CA}/\textsf{PAI}_{8}$ canonical model. Thus, if $\varphi$ is not a consequence of $\Gamma$ in $\textsf{CA}/\textsf{PAI}_{8}$, $\mathfrak {M}_{\Gamma }$ provides a right decomposable countermodel. $\square$

Next is the proof of Theorem 6 characterizing the extension $\textsf{CA}/\textsf{PAI}_{7}$:

### Proof

As in Theorem 5, soundness is easy. For completeness, let $\Gamma$ be a maximally consistent $\textsf{CA}/\textsf{PAI}_{7}$ theory. By Lemma 10, $\Gamma \vdash \textbf{t}_{(\varphi \rightarrow \psi )\wedge (\psi \rightarrow \xi )}\rightarrow \textbf{t}_{\varphi \rightarrow \xi }$.

$$\begin{aligned} \llbracket \varphi \rrbracket _{\Gamma }&\multimap _{\Gamma }\llbracket \xi \rrbracket _{\Gamma }=~ ~ \! \llbracket \varphi \rightarrow \xi \rrbracket _{\Gamma }\le _{\Gamma }\llbracket (\varphi \rightarrow \psi )\wedge (\psi \rightarrow \xi )\rrbracket _{\Gamma }\\&=(\llbracket \varphi \rrbracket _{\Gamma }\multimap _{\Gamma }\llbracket \psi \rrbracket _{\Gamma })\oplus _{\Gamma }(\llbracket \psi \rrbracket _{\Gamma }\multimap _{\Gamma }\llbracket \xi \rrbracket _{\Gamma }) \end{aligned}$$

So $\mathfrak {M}_{\Gamma }$ is a middle term eliminable model providing a counterexample in case $\Gamma \nvdash \zeta$. $\square$

Now, the proof of Theorem 7 can be given:

### Proof

Let $\Gamma$ be a maximally consistent $\textsf{CA}/\textsf{PAI}_{11}$ theory. We show that $\mathfrak {M}_{\Gamma }$ has the property. By Lemma 10, we infer that  is in $\Gamma$. It is easy to prove that  is a $\textsf{CA}/\textsf{PAI}$ theorem, which ensures that $\Gamma \vdash \textbf{t}_{\varphi \rightarrow \psi }\rightarrow \textbf{t}_{\varphi \wedge \psi }$. Consequently, $\llbracket \varphi \wedge \psi \rrbracket _{\Gamma }\le _{\Gamma }\llbracket \varphi \rightarrow \psi \rrbracket _{\Gamma }$ must hold, whence:

$$\begin{aligned} \llbracket \varphi \rrbracket _{\Gamma }\oplus _{\Gamma }\llbracket \psi \rrbracket _{\Gamma }=\llbracket \varphi \wedge \psi \rrbracket _{\Gamma }\le _{\Gamma }\llbracket \varphi \rightarrow \psi \rrbracket _{\Gamma }=\llbracket \varphi \rrbracket _{\Gamma }\multimap _{\Gamma }\llbracket \psi \rrbracket _{\Gamma } \end{aligned}$$

As $\varphi$ and $\psi$ were arbitrary, this holds for any pair of elements in $\mathcal {T}_{\Gamma }$. $\square$

Finally, we present the proof of Theorem 8 in which the demodalized $\textsf{CA}/\textsf{DAI}$ is characterized:

### Proof

Let $\Gamma$ be a maximally consistent $\textsf{CA}/\textsf{DAI}$ theory. We show that the accessibility relation of its canonical model $\mathfrak {M}_{\Gamma }$ is diagonal by demonstrating that $\Gamma ^{\Box }=\Gamma$ for arbitrary $\Gamma$.

- For $\Gamma ^{\Box }\subseteq \Gamma$, pick a $\varphi \in \Gamma ^{\Box }$. Then $\textbf{t}_{\varphi }\rightarrow \varphi \in \Gamma$ and $\textbf{t}_{\varphi }\in \Gamma$, whence $\varphi \in \Gamma$.
- For $\Gamma \subseteq \Gamma ^{\Box }$, pick a $\varphi \in \Gamma$. As , $\Gamma \vdash \textbf{t}_{\varphi }\rightarrow \varphi$, whence $\varphi \in \Gamma ^{\Box }$.

So, by maximality of $\Gamma$, if $\Gamma R\Delta$, $\Delta =\Gamma$. $\square$

## Appendix D: Decidability of $\textsf{CA}/\textsf{PAI}$

As Fine’s (1986), Dunn’s (1972), and Deutsch’s (1985) show, the method of filtrations ports easily to the case of logics in the constellation of $\textsf{PAI}$. In particular, Deutsch’s proof of decidability of his $\textsf{S}$ carries over with only minor modifications; to maximize the utility of the shared material, we will retain much of Deutsch’s notation and idioms. For the first component of a proof through filtrations:

### Definition 21

For a $\textsf{CA}/\textsf{PAI}$ model, formula $\varphi$, and set $\Gamma _{\varphi }$ of subformulae of $\varphi$, define a relation $\approx _{\varphi }$ so that

$$\begin{aligned} w\approx _{\varphi }w'\;\text {if for all}\; \psi \in \Gamma , (w\Vdash \psi \;\text {iff}\;w'\Vdash \psi ) \end{aligned}$$

*I.e.* $\approx _{\varphi }$ identifies worlds when they agree with respect to the subformulae of $\varphi$. That $\approx _{\varphi }$ is an equivalence relation follows from standard reasoning; let $|w|_{\varphi }$ be the equivalence class of *w* on the set of worlds in the model.

### Definition 22

For a model, define $R_{\varphi }$ so that $|w|_{\varphi }R_{\varphi }|w'|_{\varphi }$ holds if for all $\psi \rightarrow \xi \in \Gamma _{\varphi }$, if $w\Vdash \psi \rightarrow \xi$ then $w'\Vdash \psi \rightarrow \xi$.

The following facts may be justified along similar lines as Deutsch (1985):

### Lemma 21

The following hold:

- $R_{\varphi }$ is transitive and reflexive
- if $$wRw'$$ then $$|w|_{\varphi }R_{\varphi }|w'|_{\varphi }$$
- if $|w|_{\varphi } R_{\varphi }|w'|_{\varphi }$, $w\approx _{\varphi }u$, and $w'\approx _{\varphi }u'$ then $|u|_{\varphi } R_{\varphi }|u'|_{\varphi }$.

### Definition 23

For a $\textsf{CA}/\textsf{PAI}$ model, formula $\varphi$, and set $\Gamma _{\varphi }$ of subformulae of $\varphi$, let $\equiv _{w}$ be introduced so that

$$\begin{aligned} \psi \equiv _{w}\xi \;\text {if whenever}\;w\approx _{\varphi }w'\;\text {then}\; t_{w'}(\psi )=t_{w'}(\xi ) \end{aligned}$$

The equivalence class of a formula $\psi$ under $\equiv _{w}$ will be denoted by $[\psi ]_{w}$. This yields a further definition:

### Definition 24

$${[}\psi ]_{w}^{\varphi }={\left\{ \begin{array}{ll}_{w}\hbox { if }\psi \notin \Gamma _{\varphi }\\ {[}\psi ]_{w}\cap \Gamma _{\varphi } \text{ otherwise }.\end{array}\right. }$$

Note that as $\Gamma _{\varphi }$ is finite, $[\psi ]_{w}^{\varphi }$ will be finite whenever $\psi \in \Gamma _{\varphi }$.

### Definition 25

For such a model, we define

- $$[\psi ]_{w}^{\varphi }\oplus _{|w|}[\xi ]_{w}^{\varphi }=[\psi \wedge \xi ]_{w}^{\varphi }$$
- $$[\psi ]_{w}^{\varphi }\multimap _{|w|}[\xi ]_{w}^{\varphi }=[\psi \rightarrow \xi ]_{w}^{\varphi }$$.

Deutsch’s remarks in Deutsch (1985) carry over without modification to establish that $\oplus _{|w|}$ is idempotent, commutative, and associative; with trivial modifications, his remarks establish that $\oplus _{|w'|_{\varphi }}$ and $\multimap _{|w'|_{\varphi }}$ are functions. We then can define the filtration:

### Definition 26

For a $\textsf{CA}/\textsf{PAI}$ model $\mathfrak {M}$ and formula $\varphi$, let $\mathfrak {M}_{\varphi }$ be defined so that

- $$W_{\varphi }=W^{\approx _{\varphi }}$$
- for each $$|w|_{\varphi }\in W_{\varphi }$$, $$\mathcal {T}_{|w|_{\varphi }}=\lbrace [\psi ]_{w}^{\varphi }\mid \psi \in \mathcal {L}\rbrace $$
- for each $|w|_{\varphi }\in W_{\varphi }$, $t_{|w|}(p)=[p]_{w}^{\varphi }$ for atoms *p*
- $$v^{\varphi }(p)=\lbrace |w|_{\varphi }\mid p\in \Gamma _{\varphi } \text{ and } \mathfrak {M},w\Vdash p\rbrace $$

and $R_{\varphi }$, $\oplus _{|w|_{\varphi }}$ and $\multimap _{|w|_{\varphi }}$ are drawn from the earlier definitions.

The same inductions Deutsch uses in Deutsch (1985) port almost directly to establish the following:

### Lemma 22

The following hold:

- $$\mathfrak {M}_{\varphi }$$ is a $$\textsf{CA}/\textsf{PAI}$$ model
- $$t_{|w|_{\varphi }}^{\varphi }(\psi )=[\psi ]_{w}^{\varphi }$$ for all $$\psi $$.

As the negation in $\textsf{CA}/\textsf{PAI}$ is Boolean, the following lemma is easier in the present case than for Deutsch’s paraconsistent $\textsf{S}$. Deutsch’s clauses for verification (in Deutsch’s notation, that $\textbf{t}$ is assigned to $\psi$) directly apply, making negation the only novel element to its proof.

### Lemma 23

For all $\psi \in \Gamma _{\varphi }$, $\mathfrak {M}_{\varphi },|w|_{\varphi }\Vdash \psi$ iff $\mathfrak {M},w\Vdash \psi$.

### Proof

The basis step is by definition and continues by induction on complexity of $\psi$. As negation is classical, that $\mathfrak {M}_{\varphi },|w|_{\varphi }\Vdash \lnot \xi$ iff $\mathfrak {M},w\Vdash \lnot \xi$ follows from the induction hypothesis that $\mathfrak {M}_{\varphi },|w|_{\varphi }\nVdash \xi$ iff $\mathfrak {M},w\nVdash \xi$. All other steps follow by recasting the clauses for verification as clauses about truth at $|w|_{\varphi }$ and *w*. $\square$

As in Deutsch (1985), we cannot ensure that the topic semilattices are finite but can borrow Deutsch’s analogous (and sufficient) property of a model’s being *finite for a sentence* $\varphi$, i.e., that *W* is finite while for any $\psi ,\xi \in \Gamma _{\varphi }$, the sets $t_{|w|_{\varphi }}(\psi )\oplus _{|w|_{\varphi }}t_{|w|_{\varphi }}(\xi )$ and $t_{|w|_{\varphi }}(\psi )\multimap _{|w|_{\varphi }}t_{|w|_{\varphi }}(\xi )$ are finite. It turns out that this is enough.

### Lemma 24

Every $\varphi$ not provable in $\textsf{CA}/\textsf{PAI}$ has a countermodel that is finite for $\varphi$.

### Proof

By modifying Deutsch’s proof to add a clause inferring finiteness of $t_{|w|_{\varphi }}(\psi )\multimap _{|w|_{\varphi }}t_{|w|_{\varphi }}(\xi )$ from its identity with the finite set $[\psi \rightarrow \xi ]_{|w|_{\varphi }}^{\varphi }$. $\square$

As the set of worlds and the number of points in the topic semilattice to be surveyed are finite, this means that the nontheorems of $\textsf{CA}/\textsf{PAI}$ are recursively enumerable. As theorems, too, are recursively enumerable, we are able to infer Theorem 4.

## Footnotes

1 Indeed, some of the concluding remarks in Fine (1986) bring up the question of whether *any* logical connectives are transparent in this sense.

2 Technically, Fine (1986), too, provides model theory for an *extension* of Parry’s logic, carrying over an axiom introduced by Dunn (1972). But the addition is very modest and natural and the resulting system is frequently treated as a sort of “completion” of the axioms in Parry (1933); indeed, the Dunn–Fine axiom is ultimately endorsed by Parry (1989).

3 Fine’s model theory from Fine (1986) uses the notation “$\langle \mathcal {I},\circ \rangle$” and the topic assignment function is denoted by “$\gamma$”; our choice of notation maintains continuity with Berto’s model-theoretic presentation of TSIMs.

4 *[D]er Begriff des logischen Folgens die angegebene Eigenschaft haben, da die Konklusion keine anderen Begriffe enthalten kann als die Prämissen.*

5 This system is clearly intermediate between Parry’s $\textsf{PAI}$ and the weak system $\textsf{CA}/\textsf{PAI}$ to be introduced. One roadblock to quickly axiomatizing $\textsf{R}/\textsf{PAI}$ is the difficulty of representing these elements $\mathfrak {i}_{w}$ in the canonical model as there is no particular element of syntax that naturally corresponds to $\mathfrak {i}_{w}$.

6 As suggested in Sect. 2.2, the axiom [*A*11] corresponds to the property of subtopic preservation, which will be demonstrated in Sect. 4.4.

7 This also demonstrates that $\textsf{CA}/\textsf{PAI}$ is in fact a subsystem of $\textsf{PAI}$.

8 Thanks to Francesco Berto for suggesting an acknowledgment of this feature.

9 Context should avoid confusion between this use and the use of the symbol for semilattice join.
